# Comparative tetracycline removal by *Acacia mearnsii*-derived carbons and a cellulose-derived iron oxide/oxyhydroxide carbon composite

**DOI:** 10.1039/d6ra06684d

**Published:** 2026-09-01

**Authors:** Elham Jalali, Elizabeth Erasmus, Shahab Maghsoudi, Marietjie Schutte-Smith, Ivan Novak, Hendrik. G. Visser

**Affiliations:** a Chemistry Department, University of the Free State Bloemfontein 9300 South Africa visserhg@ufs.ac.za; b Department of Chemistry, Faculty of Science, Shahid Bahonar University of Kerman Kerman Iran; c Pod Rovnicami 7 84104 Bratislava Slovakia

## Abstract

Tetracycline (TC) contamination of aquatic systems poses severe ecological and public health challenges. This study compares four bio-derived carbon adsorbents utilizing targeted iron-functionalization strategies to optimize antibiotic decontamination: (A) thermally modified *Acacia mearnsii* bark extract, (B) *A. mearnsii* carbon loaded with 25% Fe(OH)_3_ (acidic precipitation), (C) *A. mearnsii* carbon loaded with 25% Fe(OH)_3_ (NH_4_OH-adjusted), and (D) a delignified cellulose-derived carbon-fibre composite bearing surface iron oxide/oxyhydroxide phases. Nitrogen physisorption revealed a transition from mesoporous architectures (Type IV(a), Sample A) to highly microporous frameworks (Type I(a), Sample D), with *t*-plot micropore volumes increasing from 0.032 to 0.102 cm^3^ g^−1^. High-resolution Fe 2p XPS confirmed that the surface iron species are predominantly Fe(iii) (hydr)oxides. Non-linear kinetic modelling of the 20–120 min uptake data showed that the pseudo-first-order model provided the best statistical description for all samples (*R*^2^ = 0.950–0.995). The fitted equilibrium capacities ranged from 6.57 to 9.48 mg g^−1^, remaining within the 10 mg g^−1^ mass-balance limit imposed by the kinetic conditions (*C*_0_ = 10 mg L^−1^; dosage = 1.0 g L^−1^). Intraparticle-diffusion analysis indicated that diffusion contributed to TC uptake but was not the sole rate-controlling process. Under optimized batch parameters (1.0 g L^−1^, *C*_0_ = 10 mg L^−1^, 25 °C), maximum TC removal efficiencies were 92.7% at pH 7.0 (A), 88.7% at pH 5.0 (B), 84.6% at pH 5.0 (C), and 90.0% at pH 7.0 (D). Equilibrium data were best described by the Freundlich model for Sample A (*R*^2^ = 0.997) and by the Langmuir model for Samples B–D. The fitted Langmuir capacities of 20.3–20.8 mg g^−1^ were obtained from the isotherm dataset collected at initial TC concentrations of 10–40 mg L^−1^ and should not be interpreted as capacities measured under the separate fixed-concentration kinetic condition of *C*_0_ = 10 mg L^−1^. Sample D achieved 77.5 ± 0.5% TC removal efficiency in the fourth regeneration cycle, corresponding to 86.5% relative retention of its first-cycle removal efficiency. This four-cycle result provides preliminary laboratory-scale evidence of reusability under the tested conditions.

## Abstract 2

Tetracycline (TC) contamination of aquatic systems poses severe ecological and public health challenges.

## Graphical abstract

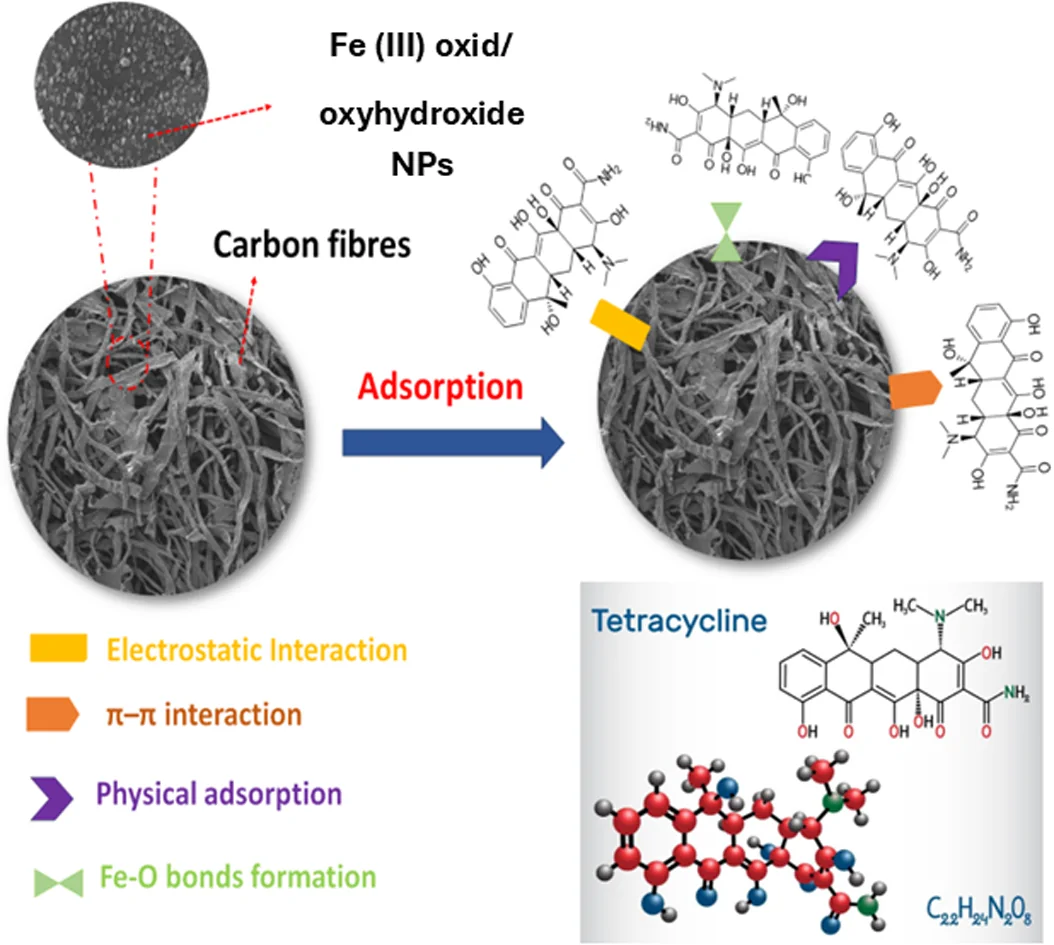

## 1. Introduction

The pervasive contamination of aquatic environments by pharmaceutical residues, particularly broad-spectrum antibiotics, presents a critical global threat to ecotoxicological stability and public safety.^1^ Due to their widespread and often unmonitored use in human clinical therapies, aquaculture operations, and veterinary husbandry, large quantities of unmetabolized antibiotics are continuously discharged into municipal and industrial waste streams.^2^ Conventional wastewater treatment plants, primarily engineered for bulk organic carbon and nutrient removal, are largely ineffective at eliminating these trace recalcitrant organic micro-pollutants.^3^ Tetracycline (TC), a widely used amphoteric antibiotic, is highly resistant to natural environmental biodegradation due to its stable, highly conjugated multi-ring naphthacene structure.^4^ Chronic exposure to sub-lethal TC concentrations in natural water bodies accelerates the proliferation of antibiotic-resistant bacteria (ARB) and antibiotic-resistance genes (ARGs), potentially neutralising essential clinical therapies. Consequently, developing highly efficient, green, and economically viable remediation technologies is an urgent priority.

Among various advanced treatment options, liquid-phase adsorption is a highly efficient, scalable, and simple approach for capturing trace organics. While commercial activated carbons remain standard choices, their widespread industrial application is constrained by high production costs, energy-intensive activation processes, and low cyclical regenerability.^5–8^ This has focused research on engineering functionalized bio-adsorbents using abundant, non-toxic agricultural and industrial byproducts. Condensed tannins extracted from *Acacia mearnsii* (Black wattle) bark represent an excellent alternative precursor network. Rich in polyflavanoid architectures composed of flavan-3-ol subunits (including catechin, gallocatechin, fisetinidol, and robinetinidol), this biopolymer contains a high density of *ortho*-dihydroxyphenolic (catechol) groups capable of strong coordination with transition metal ions.^9,10^ Integrating iron oxide nanoparticles or hydrous iron frameworks onto carbonaceous supports creates highly responsive hybrid composites. The underlying carbon skeleton provides a porous structural platform that prevents nanoparticle aggregation, while the immobilized iron species introduce high-density Lewis acid centres capable of highly selective inner-sphere coordination with electron-donating groups on the antibiotic structure. Crucially, the incorporation of surface Fe(iii) oxide/oxyhydroxide phases imparts additional reactivity to the material, which can enhance its downstream chemical recyclability and active site performance.^11–14^

Through a systematic comparison of structural, surface-chemical, and adsorption behaviour across the four adsorbents, we analyze the correlations between structural configurations, surface charging profiles, iron speciation, and TC uptake dynamics. Advanced spectroscopic and physisorption validations are used to detail the multi-pathway adsorption mechanisms and evaluate the regeneration behaviour of these composites over four adsorption–desorption cycles. Moreover, the incorporation of iron oxide/oxyhydroxide-carbon composite imparts catalytic properties to the material, enhancing its recyclability and efficiency.^15^ To systematically investigate the effects of surface functionalization and iron speciation on the efficiency of TC removal, four carbon-based composite materials were prepared and comprehensively characterized: (A) thermally modified *Acacia mearnsii* extract, (B) *Acacia mearnsii* extract carbon loaded with 25 wt% Fe(OH)_3_ under acidic precipitation conditions, (C) *Acacia mearnsii* extract carbon loaded with 25 wt% Fe(OH)_3_ under NH_4_OH adjusted basic conditions, and (D) iron oxide/oxyhydroxide-carbon composite immobilized on a delignified cellulose-derived carbon fibre scaffold. The study compares adsorbents prepared from two different carbon precursors and with different iron-incorporation procedures. Consequently, the observed performance differences are interpreted as combined effects of precursor chemistry, carbonisation history, porosity, morphology, and surface iron speciation, rather than as the isolated effect of iron-loading strategy. The comparison is intended to relate measured differences in morphology, textural properties, surface chemistry, pH-dependent adsorption behaviour, and TC removal performance. Because the materials differ in precursor composition, thermal history, and iron-incorporation procedure, the observed differences are not attributed solely to iron loading or to a single synthesis variable. In this work, four compositionally distinct carbonaceous adsorbents were compared: an iron-free *Acacia mearnsii*-derived carbon (Sample A), two iron-modified *Acacia mearnsii*-derived carbons prepared under different precipitation conditions (Samples B and C), and a cellulose-derived carbon-fibre composite containing surface iron oxide/oxyhydroxide phases (Sample D). Rather than maximising absolute adsorption capacity, our aim is to elucidate how precursor configuration and surface Fe speciation control adsorption mechanisms, kinetics, regenerability, and competitive-anion tolerance during TC removal. By correlating FTIR, XPS, BET, and XRD characterisation with kinetic/isotherm modelling and competing-anion tests, we identify a cellulose-derived Fe(iii) oxide/oxyhydroxide-carbon composite (Sample D) that combines rapid adsorption, high reusability, and sensitivity to co-existing inorganic anions under laboratory electrolyte conditions.

## 2. Experimental

### 2.1 Materials

Ferrous sulphate heptahydrate (FeSO_4_·7H_2_O, analytical grade) was purchased from Centralchem (Slovakia). Sodium hydroxide (NaOH), hydrochloric acid (HCl), and ammonium hydroxide (NH_4_OH, 25%) were obtained from Mikrochem (Slovakia) and Lachner (Slovakia). High-purity tetracycline hydrochloride (98.0–102.0%, HPLC grade) was supplied by Sigma-Aldrich (St. Louis, MO, USA). Analytical-grade deionized water (18.2 MΩ cm^−1^) was used to prepare all solutions. Raw *Acacia mearnsii* bark extracts were supplied by NTE (Pietermaritzburg, South Africa), and raw delignified cellulose substrates were sourced from Slovak operations. Sorbent precursors and base materials were maintained under dry conditions by AQUA + TECH Specialities SA (Romont, Fribourg, Switzerland).

### 2.2 Adsorbent synthesis protocols

Sample A was prepared from *Acacia mearnsii* bark extract by thermal carbonisation at 400 °C without deliberate iron addition. The obtained material is referred to as the iron-free *Acacia mearnsii*-derived carbon. The carbonisation yield of Sample A ranged from 43 to 47%, calculated as (eqn (1)):1
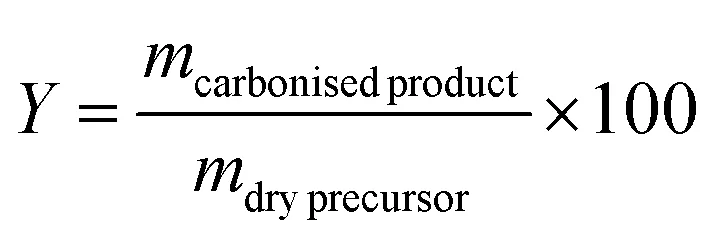
where *m*_carbonised product_ is the dry mass of the final carbonised material and *m*_dry precursor_ is the initial dry mass of *Acacia mearnsii* bark extract.

Samples B and C were prepared from thermally modified *Acacia mearnsii* bark-extract carbon using a nominal initial loading of 25 wt% Fe(OH)_3_. For Sample B, the iron-containing phase was deposited under acidic precipitation conditions. Sample C was prepared using the same nominal Fe(OH)_3_ loading but with precipitation adjusted using dilute NH_4_OH. The resulting materials are referred to as acidic-precipitated Fe(OH)_3_-modified *Acacia mearnsii* carbon (Sample B) and NH_4_OH-adjusted Fe(OH)_3_-modified *Acacia mearnsii* carbon (Sample C), respectively.

The 25 wt% value represents the nominal reagent-based iron hydroxide formulation supplied for preparation and is not treated as a measured bulk Fe concentration. The surface iron chemistry was therefore evaluated using XPS and SEM-EDS, while the absence of bulk Fe quantification is recognised as a limitation of the present study.

Sample D was synthesized *via* a dual-stage pathway involving high-temperature carbonization of a fibrous biomass template followed by *in situ* nanoparticle precipitation. In the first step, 20 kg of raw delignified cellulose microfibres (dimensions: 5–15 µm *×* 50–200 µm) were isolated in a sealed stainless–steel reactor vessel (50 cm × 130 cm *×* 60 cm) equipped with a tight-fitting lid to maintain a self-generated inert nitrogen atmosphere. The assembly was transferred into an industrial muffle furnace. The thermal profile was programmed with a multi-stage heating ramp to preserve the structural fibre framework: heating from ambient to 300 °C at 20 °C h^−1^, shifting to a slow rate of 5 °C h^−1^ between 300 °C and 400 °C to manage volatile evolution, and ramping at 20 °C h^−1^ to a maximum carbonization temperature of 700 °C, which was maintained for 10 h. Off-gases (H_2_O and CO_2_) were routed through an automated fume extraction scrubber. The reactor was allowed to cool to ambient temperature inside the furnace, yielding highly structured carbon fibre micro-scaffolds.^16,17^

In the second step, 247 g of FeSO_4_·7H_2_O, was dissolved in 4000 mL of deionized water inside a 10 L stirred batch reactor. Then, 200 g of the carbonized microfibres were dispersed into the iron electrolyte under constant paddle stirring (150 rpm) until a homogeneous slurry was achieved. *In situ* precipitation of surface-bound iron phases was initiated by adding an alkaline solution (72 g NaOH dissolved in 3000 mL deionized water). The addition rate was controlled to maintain a solution pH 8.0, driving the formation and precipitation of uniform Fe(OH)_2_ layers onto the carbon fibre matrices. The slurry was stirred for 4 h, filtered, and washed repeatedly with deionized water until the effluent reached neutrality (pH 7.0–8.0) to stabilize the mineral deposit against premature leaching. The filter cake was dried at 100 °C to a constant mass, yielding approximately 280 g of composite with a nominal reagent-based formulation corresponding to approximately 28 mass% Fe(OH)_2_ precursor. This value was not verified as a final bulk Fe concentration. The final thermal treatment was intended to stabilise the deposited iron-containing precursor on the carbon-fibre scaffold. Based on the Fe 2p XPS results, the surface iron is described in this study as predominantly Fe(iii) oxide/oxyhydroxide species. The available characterisation does not establish the presence of metallic Fe(0).

The adsorbents in this study were not prepared as a fully factorial control series. In particular, Sample D was prepared from a cellulose precursor using a carbonisation procedure distinct from that used for the *Acacia mearnsii*-derived materials. Therefore, the results are interpreted as a comparison among compositionally and structurally different materials, and performance differences cannot be assigned solely to the iron-incorporation procedure.

### 2.3 Batch adsorptive testing

A concentrated aqueous stock solution of TC (1000 mg L^−1^) was prepared by dissolving TC in deionised water. Working solutions of 10 mg L^−1^ were obtained by serial sequential dilution with deionised water for batch adsorption experiments. For isotherm studies, six initial TC concentrations of 10, 15, 20, 25, 30, and 40 mg L^−1^ were prepared by serial dilution from the stock solution; these correspond to equilibrium concentrations (Ce) spanning 0.6–22.3 mg L^−1^ at a dosage of 1.0 g L^−1^, covering the rising portion of the isotherm curve and approaching the saturation plateau.

Batch experiments were performed in series by adding a predetermined adsorbent dosage (0.6–5.0 g L^−1^) to 10 mL of TC solution in sealed Erlenmeyer flasks. The mixtures were agitated at 200 rpm in a temperature-controlled water bath at 25 ± 1 °C. The solution pH was systematically adjusted over a range of 3.0–11.0 using 0.1 M HCl or NaOH.

Contact-time experiments were conducted from 20 min to 24 h to evaluate the evolution of TC removal under the selected batch conditions. Calibration curves were constructed using standard TC solutions in the concentration range of 0.015–50 mg L^−1^. Following equilibration, the suspensions were filtered through 0.45 µm membrane filters, and the residual TC concentration in the filtrate was determined using an Agilent Cary 60 UV-vis spectrophotometer at 357 nm. Because TC absorbance at 357 nm varied with solution pH, pH-specific calibration curves were prepared at nominal pH 3, 5, 7, 9, and 11 over the concentration range of 0.015–10.0 mg L^−1^ (Table S6, SI). The pH-dependent adsorption samples were quantified using the calibration curve corresponding to the nearest nominal pH condition. Membrane-filter recovery, matrix spike-recovery, adsorbent-blank absorbance, limits of detection, and limits of quantification are summarised in Table S7 (SI). Initial and final pH values of the TC adsorption solutions are reported in Table S8 (SI). The adsorption capacity (*q*_e_, mg g^−1^) and removal efficiency (*R*(%)) were calculated using eqn (2) and (3), respectively.2
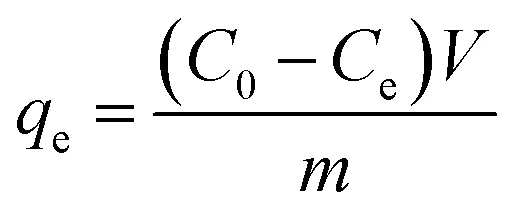
3
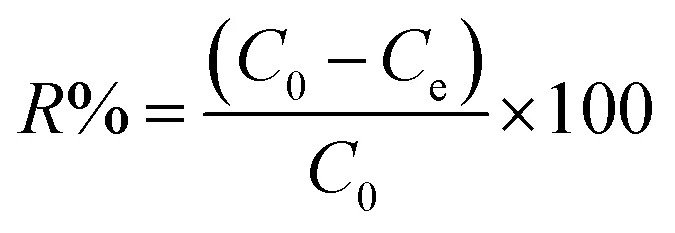
where *C*_0_ and *C*_e_ are the initial and final equilibrium concentrations of TC (mg L^−1^), *V* is the total batch solution volume (*V* = 0.010 L, *i.e.* 10 mL), and *m* is the absolute dry mass of the added sorbent composite (g) (*m* = dosage × *V* for batch optimisation experiments).

In this study, TC removal refers to the decrease in UV-vis-determined TC concentration in the filtered aqueous phase. No degradation products, total organic carbon, mineralisation, or released TC after regeneration were measured; therefore, the results do not demonstrate TC degradation or mineralisation.

For all isotherm experiments, the solution volume was 10.0 mL and the adsorbent dosage was 1.0 g L^−1^, corresponding to an adsorbent mass of 0.010 g per batch. Each condition was analysed in three independent replicates. The equilibrium contact time and solution pH were selected according to the experimentally determined optimum conditions for each adsorbent. All adsorption experiments were conducted using synthetic TC solutions prepared in deionized water under laboratory batch conditions. The present study did not include real wastewater, natural-water matrices, mixed-electrolyte systems, or continuous-flow adsorption experiments. Therefore, the results are interpreted as comparative batch adsorption data and are not used to predict full-scale water-treatment performance.

The initial pH of each TC solution was adjusted to 3.0, 5.0, 7.0, 9.0, or 11.0 before adsorbent addition. Following the selected contact time, the suspensions were filtered through 0.45 µm membrane filters and the final pH of the filtrates was measured. Tetracycline concentrations were determined at 357 nm using calibration curves prepared at the nearest corresponding nominal pH condition (pH 3, 5, 7, 9, or 11). The final pH values remained within −0.28 to +0.32 pH units of their respective initial pH values.

For the preliminary added-salt experiment, NaCl, Na_2_SO_4_, and NaNO_3_ were separately added at a nominal concentration of 20 mg L^−1^, together with an anion-free control. The salts were evaluated under the respective selected adsorption conditions for Samples A–D. The experiment was designed as a nominal-mass-concentration screening test and did not employ equal molar concentrations or matched ionic strengths. Initial solution pH was adjusted to the selected condition; final solution pH was not measured for the original added-salt experiment.

#### 2.3.1 Isotherm and kinetic model evaluation

The adsorption equilibrium data obtained from batch experiments were analysed using the Langmuir and Freundlich isotherm models to describe the interaction between TC molecules and the adsorbent surface.

The Langmuir isotherm model assumes monolayer adsorption on a homogeneous surface with finite identical binding sites and was applied to evaluate the maximum adsorption capacity (*q*_m_) and adsorption affinity (*K*_L_). The Freundlich isotherm model, which assumes heterogeneous surface adsorption and multilayer formation, was used to describe adsorption intensity and surface heterogeneity through the constants *K*_F_ and *n*.

Equilibrium isotherm experiments were conducted using initial TC concentrations of 10, 15, 20, 25, 30 and 40 mg L^−1^. Each batch contained 10.0 mL of TC solution and 0.010 g of adsorbent, corresponding to an adsorbent dosage of 1.0 g L^−1^. Experiments were conducted at 25 ± 1 °C and 200 rpm, using the optimum pH and experimentally determined equilibrium contact time for each adsorbent. Equilibrium adsorption capacity was calculated from *q*_e_ = (*C*_0_ − *C*_e_)*V*/*m*. The nonlinear Langmuir and Freundlich equations were fitted directly to the untransformed *q*_e_-*versus-C*_e_ data. Under the isotherm conditions, the mass-balance maximum adsorption capacity depended on the initial TC concentration because the adsorbent mass and solution volume were fixed at 0.010 g and 0.010 L, respectively. Accordingly, the theoretical batch maximum was 10, 15, 20, 25, 30, or 40 mg g^−1^ for initial TC concentrations of 10, 15, 20, 25, 30, or 40 mg L^−1^, respectively. These concentration-dependent limits are distinct from the 10 mg g^−1^ maximum applicable to the kinetic experiments conducted only at *C*_0_ = 10 mg L^−1^.

Adsorption kinetic data used for kinetic modelling were collected at contact times of 20, 30, 40, 50, 60, 70, 80, 90, 100, 110, and 120 min under the selected operating conditions (*C*_0_ = 10 mg L^−1^, *V* = 10.0 mL, adsorbent dosage = 1.0 g L^−1^, and 25 ± 1 °C). The maximum physically attainable adsorption capacity under these conditions was 10 mg g^−1^, calculated from *q*_max,batch_ = *C*_0_*V*/*m*. The pseudo-first-order (PFO), pseudo-second-order (PSO), and Weber–Morris intraparticle-diffusion (IPD) models were fitted directly to the untransformed *q*_*t*_-*versus*-time data by non-linear least-squares regression for PFO and PSO, and linear regression for the IPD plot. Model selection was based on the coefficient of determination, adjusted *R*^2^, residual sum of squares, reduced chi-square values, parameter uncertainty, and physical consistency of the fitted parameters with the batch mass balance. A better empirical kinetic fit was not interpreted as direct proof of a specific molecular adsorption mechanism.

Isotherm parameters were obtained by nonlinear regression of the untransformed equilibrium data. Model selection was based on adjusted *R*^2^, reduced *χ*^2^, SSE and the physical plausibility of the fitted parameters. Kinetic model fitting is described separately in Section 3.7. For all pH, dosage and contact-time experiments at *C*_0_ = 10 mg L^−1^, equilibrium capacities *q*_e_ and dynamic capacities *q*_*t*_ were recalculated from *q* = (*C*_0_ − *C*)*V*/*m* using *V* = 0.010 L and the actual sorbent mass *m*, and were verified to satisfy *q* ≤ *C*_0_*V*/*m* for each dosage.

### 2.4 Physicochemical characterisation

Surface functional groups of the prepared materials were analysed using Fourier-transform infrared (FTIR) spectroscopy (Bruker Tensor 27, Germany) equipped with a single-bounce diamond attenuated total reflectance (ATR) accessory. Spectra were recorded and processed using OPUS v1.1 software.

Surface morphology and microstructural features were examined using field-emission scanning electron microscopy (FE-SEM) (JSM-7800F, JEOL, Japan). Elemental composition was determined using energy-dispersive X-ray spectroscopy (EDX) coupled to the SEM system.

Thermal stability and decomposition behaviour were evaluated using thermogravimetric analysis (TGA) (Mettler-Toledo TGA/SDTA851, Switzerland). Approximately 5 mg of each sample was heated from room temperature to 700 °C at a heating rate of 10 °C min^−1^ under a nitrogen atmosphere (flow rate: 75 mL min^−1^). The instrument was stabilised at 25 ± 1 °C for 30 min prior to analysis. Data were processed using STAR SW 8.10 software, and the experimental error was estimated to be approximately ±5%.

Bulk Fe content was not determined by ICP-OES, ICP-MS, or XRF. Accordingly, the present study uses XPS to assess the surface chemical state of Fe and SEM-EDS to confirm the local presence of Fe-containing phases. These techniques do not provide a quantitative total Fe loading and are not used to compare bulk Fe concentrations among Samples B–D.

#### 2.4.1 Point of zero charge (pH_pzc_) and zeta potential determination

The point of zero charge (pH_pzc_) was determined using the pH drift method. Briefly, 50 mL of 0.01 M NaCl solution was prepared, and the initial pH (pH_i_) was adjusted between 2 and 12 using 0.1 M HCl or NaOH. Subsequently, 0.1 g of adsorbent was added to each solution. The suspensions were sealed and agitated at 200 rpm for 24 h at 25 ± 1 °C. After equilibration, the final pH (pH_f_) was measured, and ΔpH (pH_f_ − pH_i_) was calculated. The pH_pzc_ was identified as the pH at which ΔpH equals zero.

Zeta potential measurements were performed using a Malvern Zetasizer. Sorbent dispersions (0.05 g L^−1^) were prepared in 0.01 M NaCl solution and sonicated for 15 min to ensure uniform dispersion. The pH was adjusted over a range of 2 to 11 using dilute HCl or NaOH. Samples were equilibrated for 2 h at 25 ± 1 °C prior to analysis. Each measurement was performed in triplicate. The isoelectric point (IEP) was defined as the pH at which the zeta potential equals zero.

X-ray Photoelectron Spectroscopy (XPS) measurements were performed on a Versa Probe 5000 to determine surface speciation, analysing core-level binding energies (C 1s, O 1s, N 1s, and Fe 2p) using Shirley background subtractions and mixed Gaussian–Lorentzian fitting algorithms.

## 3. Results and discussions

### 3.1 Surface chemistry and structural

**Fig. 1:**
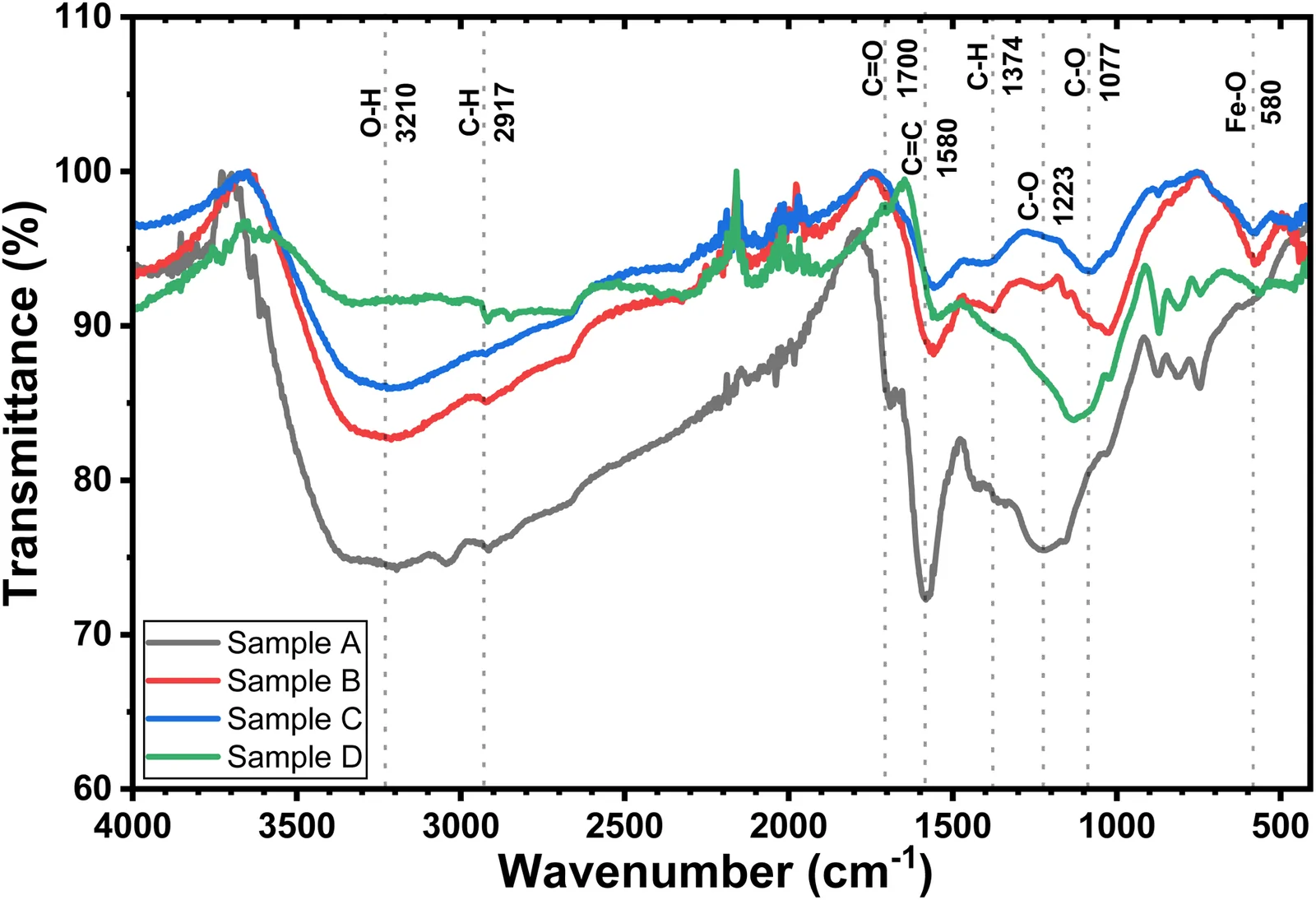
FTIR spectra of the prepared adsorbents: (A) iron-free *Acacia mearnsii*-derived carbon; (B) *A. mearnsii*-derived carbon prepared using a nominal 25 wt% Fe(OH)_3_ formulation under acidic precipitation conditions; (C) *A. mearnsii*-derived carbon prepared using a nominal 25 wt% Fe(OH)_3_ formulation with NH_4_OH adjustment; and (D) cellulose-derived carbon-fibre composite containing surface Fe(iii) oxide/oxyhydroxide phases.

FTIR spectroscopy (Fig. 1) was used to identify surface functional groups and evaluate chemical changes induced by thermal treatment and iron modification of the *Acacia mearnsii*-derived carbonaceous materials. A comparative analysis of the spectra reveals clear differences in surface chemistry across the four prepared samples.

Sample A (thermally modified *Acacia mearnsii* extract) exhibits a broad O–H stretching band centered at approximately 3430 cm^−1^, attributed to hydrogen-bonded surface hydroxyl groups and adsorbed water, consistent with the condensed polyphenolic character of the precursor. A broad absorption in the 1700–1600 cm^−1^ region reflects overlapping contributions from carbonyl (C=O) and aromatic C=C vibrations, indicating the presence of oxygenated aromatic structures within a partially graphitized carbon matrix. Additional bands at around 1400 cm^−1^ and 1040 cm^−1^ are associated with C–H bending and C–O stretching vibrations, respectively, supporting the retention of oxygenated phenolic functionalities derived from *Acacia mearnsii* proanthocyanidins.

For the iron-modified samples (Samples B and C), a noticeable reduction in the relative intensity of the oxygen-containing functional group bands (particularly in the carbonyl and C–O regions) is observed, suggesting partial transformation or complexation of these surface functionalities during thermal treatment and iron incorporation. The aromatic C=C band near 1580 cm^−1^ remains evident, indicating retention of the underlying carbonaceous framework. These changes reflect progressive modification of surface chemistry upon iron incorporation and thermal processing, consistent with the generation of Fe-oxygen coordination environments on the sorbent surface.

Sample D, based on a cellulose-derived carbon fibre scaffold with immobilised Iron oxide/oxyhydroxide surface phases, displays similar aromatic and oxygenated features but additionally exhibits a distinct band in the region 580–600 cm^−1^.^18–22^ This band is attributed to Fe–O stretching vibrations, confirming the successful incorporation of iron oxide/oxyhydroxide species within the carbonaceous matrix and supporting the XPS evidence for surface iron enrichment.

**Fig. 2:**
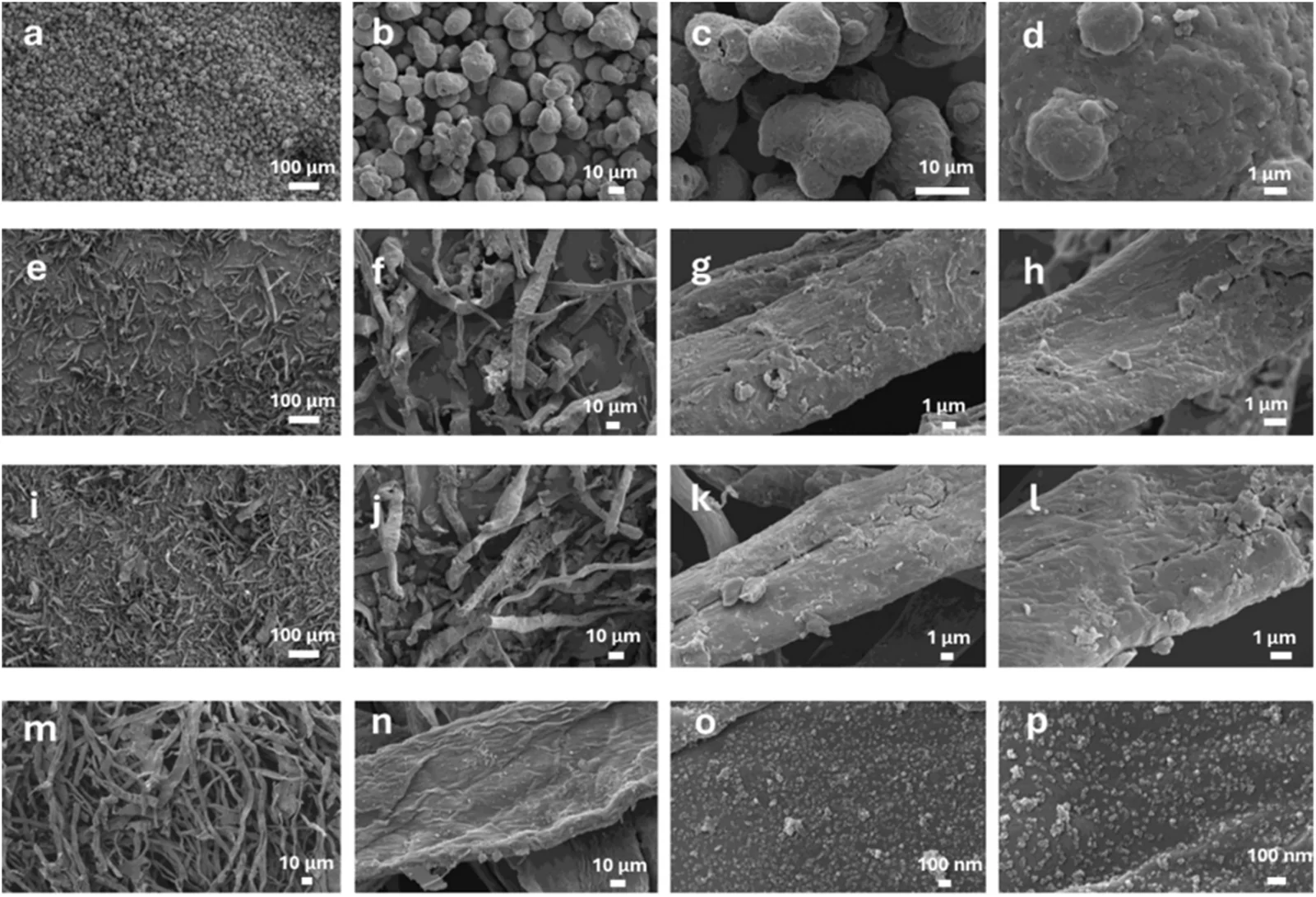
SEM micrographs of Samples A–D at different magnifications. For Sample D, discrete Fe-containing surface features are visible on the cellulose-derived carbon fibres. SEM imaging does not provide particle-size distribution or definitive Fe-phase identification.

FE-SEM micrographs (Fig. 2a–p) further reveal significant morphological evolution across the prepared materials. Sample A (Fig. 2–d) consists of irregular, aggregated globular carbon structures with rough surfaces and visible microcavities, consistent with thermal decomposition of the biomass precursor. Particle size analysis indicates an average diameter of approximately 12.8 ± 5.8 µm, with a broad size distribution. The full particle size distribution for Sample A is shown in Fig. S1 (SI).

Samples B (Fig. 2e–h) and C (Fig. 2i–l) display a transition from compact globular aggregates toward more structured and partially developed fibrous morphologies. In these samples, iron-containing phases appear dispersed across the carbon matrix, with Sample C (prepared under NH_4_OH-adjusted conditions) showing comparatively more uniform dispersion than Sample B. This suggests that synthesis conditions influence the distribution of iron species and the resulting microstructural organisation.

Sample D (Fig. 2m–p) exhibits a well-defined fibrous carbon network derived from the cellulose template, with discrete Fe-containing surface features visible along the fibre surfaces. SEM-EDS supports the local presence of Fe-associated phases; however, particle size, crystallinity, and detailed Fe-phase identity were not determined by TEM or other nanoscale characterisation methods. This morphology suggests effective immobilisation of iron species on the carbon fibre scaffold, which may enhance surface accessibility and stability.

EDS analysis (Fig. S2A–D, SI) confirms the elemental composition of all samples. Sample A consists primarily of carbon (∼88 wt%) and oxygen (∼12 wt%). SEM-EDS detected Fe in local analysed regions of Samples B and C, with approximate values of 5.8 and 7.0 wt%, respectively. These EDS values provide semi-quantitative local compositional information and are not interpreted as bulk Fe concentrations. Sample D shows a markedly different surface composition, reflecting the presence of iron-modified fibrous carbon, consistent with its distincts synthesis route.

### 3.2 Thermal stability and textural properties (TGA and N_2_ physisorption)

**Fig. 3:**
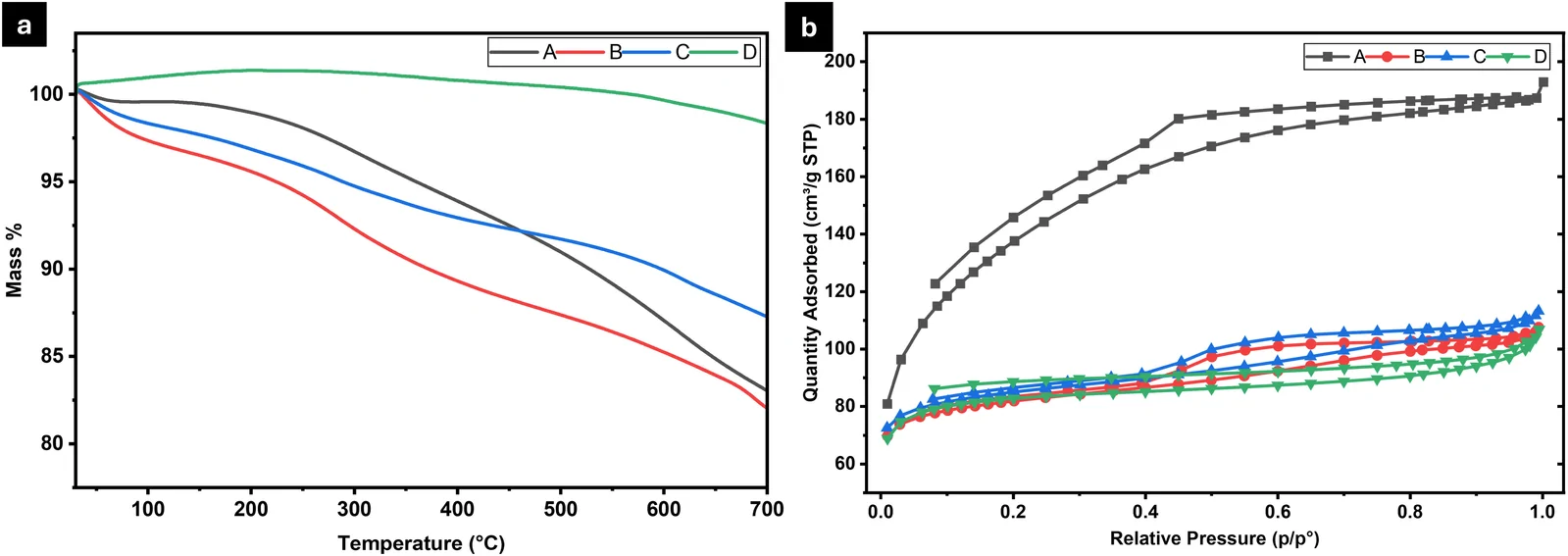
Thermal and textural characterization. (a) TGA curves (N_2_, 10 °C min^−1^) showing multi-stage decomposition for Sample A (200–306 °C), Fe(OH)_3_ dehydroxylation for Samples B and C (200–500 °C), and superior thermal stability for Sample D. (b) N_2_ physisorption isotherms (77 K) classified per IUPAC 2015: Sample A displays Type IV(a) with H3 hysteresis (*p*/*p*° = 0.4–0.95, mesoporous); Samples B and C show intermediate behaviour; Sample D exhibits Type I character (microporous, *V*_micro_ = 0.102 cm^3^ g^−1^). Filled/open symbols: adsorption/desorption.

The thermal stability and decomposition behaviour of Samples A–D were evaluated using thermogravimetric analysis (TGA), and the corresponding results are presented in Fig. 3a. The TGA curve of Sample A (thermally modified *Acacia mearnsii* extract) exhibits a multi-stage mass loss profile. An initial weight loss occurs between approximately 200–210 °C, followed by a broader degradation region between 255–306 °C, after which a stable char residue is observed at higher temperatures. This behaviour reflects the presence of structurally diverse organic constituents associated with the biomass-derived precursor, including condensed tannin and proanthocyanidin-based compounds with varying degrees of polymerisation and thermal stability.

Samples B and C, containing iron hydroxide phases, display intermediate thermal stability compared to Sample A. The observed mass loss between approximately 200 and 500 °C is attributed to the decomposition of residual organic matter combined with dehydroxylation and transformation of iron hydroxide species. The presence of iron-containing phases appears to influence the thermal decomposition pathway, resulting in a broader and more gradual mass-loss profile relative to the unmodified carbonaceous material.

In contrast, Sample D exhibits the lowest mass loss observed over the investigated temperature range, with minimal mass loss observed below 700 °C under a nitrogen atmosphere. This enhanced stability is consistent with the presence of an iron-containing carbon-fibre composite structure, where inorganic phases contribute to improved thermal resistance of the overall material.^23^

Low-temperature N_2_ physisorption analysis (77 K) was used to evaluate the textural properties of Samples A–D, and the results are summarised in Table 1 and Fig. 3b. Sample A exhibits a Type IV(a) isotherm with a well-defined H3 hysteresis loop in the relative pressure (*p*/*p*°) range of 0.40–0.95. This behaviour is characteristic of mesoporous materials with slit-shaped pores formed through particle aggregation. Sample A also presents the highest BET surface area (31.0 m^2^ g^−1^) and total pore volume (0.290 cm^3^ g^−1^), indicating a well-developed mesoporous structure.

**Table 1:** Textural properties of Samples A–D obtained from N_2_ physisorption^a^

Sample	*S*_BET_ (m^2^ g^−1^)	*V*_tot_ (cm^3^ g^−1^) (single-point desorption, high *p*/*p*_0_)	*V*_micro_ (cm^3^ g^−1^) (*t*-plot)
A	31.0	0.290	0.0318
B	11.9	0.166	0.0949
C	12.4	0.175	0.0987
D	10.3	0.165	0.102

a *S*_BET_ was obtained from BET analysis, *V*_tot_ was estimated from nitrogen adsorption at high relative pressure, and was *V*_micro_ estimated using *t*-plot analysis. BJH cumulative surface-area values are not reported because BJH analysis is not appropriate for quantitative micropore characterisation.

Upon iron incorporation (Samples B and C), a significant reduction in BET surface area is observed (11.9 and 12.4 m^2^ g^−1^, respectively), accompanied by a decrease in mesoporous contributions. This trend is consistent with partial pore blockage and structural modification associated with iron species deposition within the carbon matrix. Despite this reduction, both samples retain Type IV characteristics, indicating that mesoporosity remains present, albeit less pronounced than in Sample A.

Sample D displays a Type I-like nitrogen adsorption profile, with enhanced uptake at low relative pressure and a near-horizontal plateau at higher relative pressure. This profile is consistent with a substantial contribution from narrow pores; however, a full micropore-size distribution was not determined in the present study. Sample D exhibited the lowest BET surface area (10.3 m^2^ g^−1^) and the highest *t*-plot-derived micropore volume (0.102 cm^3^ g^−1^) among the tested materials. Because the raw adsorption/desorption data, BET fitting range, BET *C* constant, and micropore-size distribution analysis were not available for complete validation, this result is interpreted cautiously and is not used to assign adsorption performance directly to microporosity.

Overall, the materials showed substantial differences in textural characteristics. Sample A exhibited predominantly mesoporous behaviour, whereas Sample D exhibited a more microporous profile. These differences may reflect the combined effects of precursor composition, carbonisation conditions, morphology, and iron-associated surface phases. Because Sample D was prepared from cellulose using a distinct carbonisation procedure, the observed textural and adsorption differences cannot be attributed solely to iron incorporation.

A limitation of the present textural analysis is that raw adsorption/desorption isotherms, BET fitting-range diagnostics, BET *C* constants, Rouquerol consistency criteria, and an appropriate micropore-size distribution analysis were not available for re-evaluation. Therefore, the reported textural parameters are used for descriptive comparison only and not as definitive evidence for a pore-controlled TC adsorption mechanism.

### 3.3 Surface speciation *via* XPS

Surface elemental composition and chemical states were investigated using high-resolution X-ray photoelectron spectroscopy (XPS). The deconvoluted C 1s spectra (Table S2 and Fig. S3, SI) suggest that iron modification promotes the development of more ordered aromatic carbon structures following thermal treatment at 400 °C. The relative contribution of the aromatic sp^2^ C–C/C=C component (C 1, ∼284.8 eV) increases from 30.8% in Sample A to approximately 55% in Samples B and C. Sample A exhibits abundant oxygen-containing surface functionalities together with a π–π* shake-up satellite feature at 291.77 eV (2.8% of the total peak area), which is consistent with the presence of conjugated aromatic domains within the carbon matrix.

**Fig. 4:**
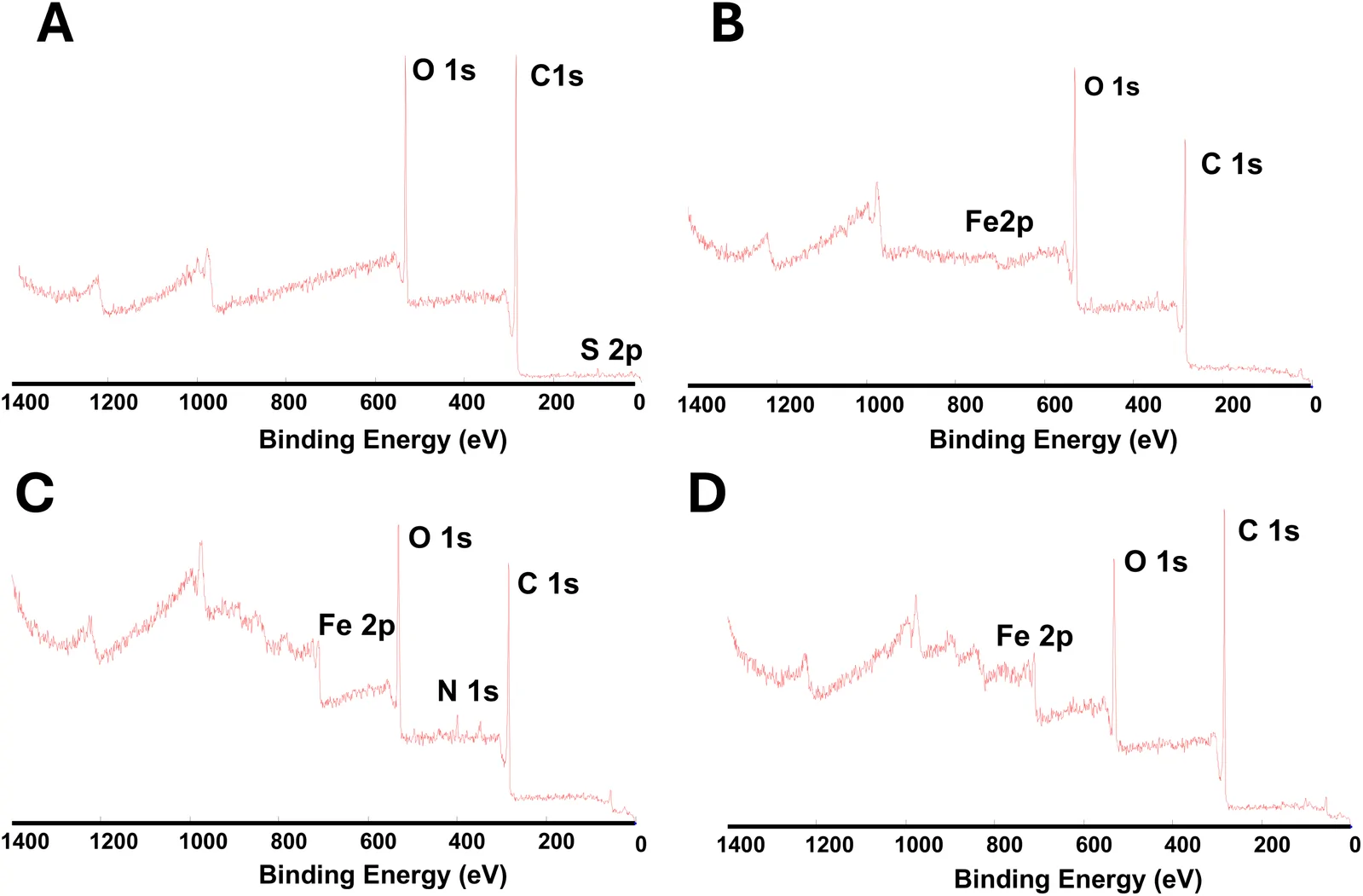
XPS analysis of Samples A–D. Survey spectra showing C 1s and O 1s peaks in all samples, with additional Fe 2p, Fe 3s, Fe 3p, and Ca 2p signals in iron-modified Samples B, C, and D. Sample C uniquely displays an N 1s peak at ∼400 eV (NH_4_^+^ from NH_4_OH synthesis).

The O 1s spectra of the iron-modified Samples B, C, and D (Fig. S4, SI) reveal a distinct low-binding-energy component (O1) centred at approximately 530.0 eV. This feature is attributed to Fe–O bonds associated with iron oxide and/or oxyhydroxide species, confirming the successful incorporation of iron-containing phases onto the carbon surface. Sample C uniquely displays an N 1s signal at approximately 400.0 eV (Fig. S5, SI), which is assigned to nitrogen-containing surface species, potentially including residual ammonium-derived functionalities originating from the NH_4_OH treatment during synthesis. Quantitative deconvolution of the O 1s region (Table S3) shows that the relative contributions of Fe–O, Fe–OH, and organic oxygen differ markedly across Samples B–D, reflecting distinct Fe(iii) oxyhydroxide speciation and organic functional group densities for the different synthesis routes (Fig. 4).

**Fig. 5:**
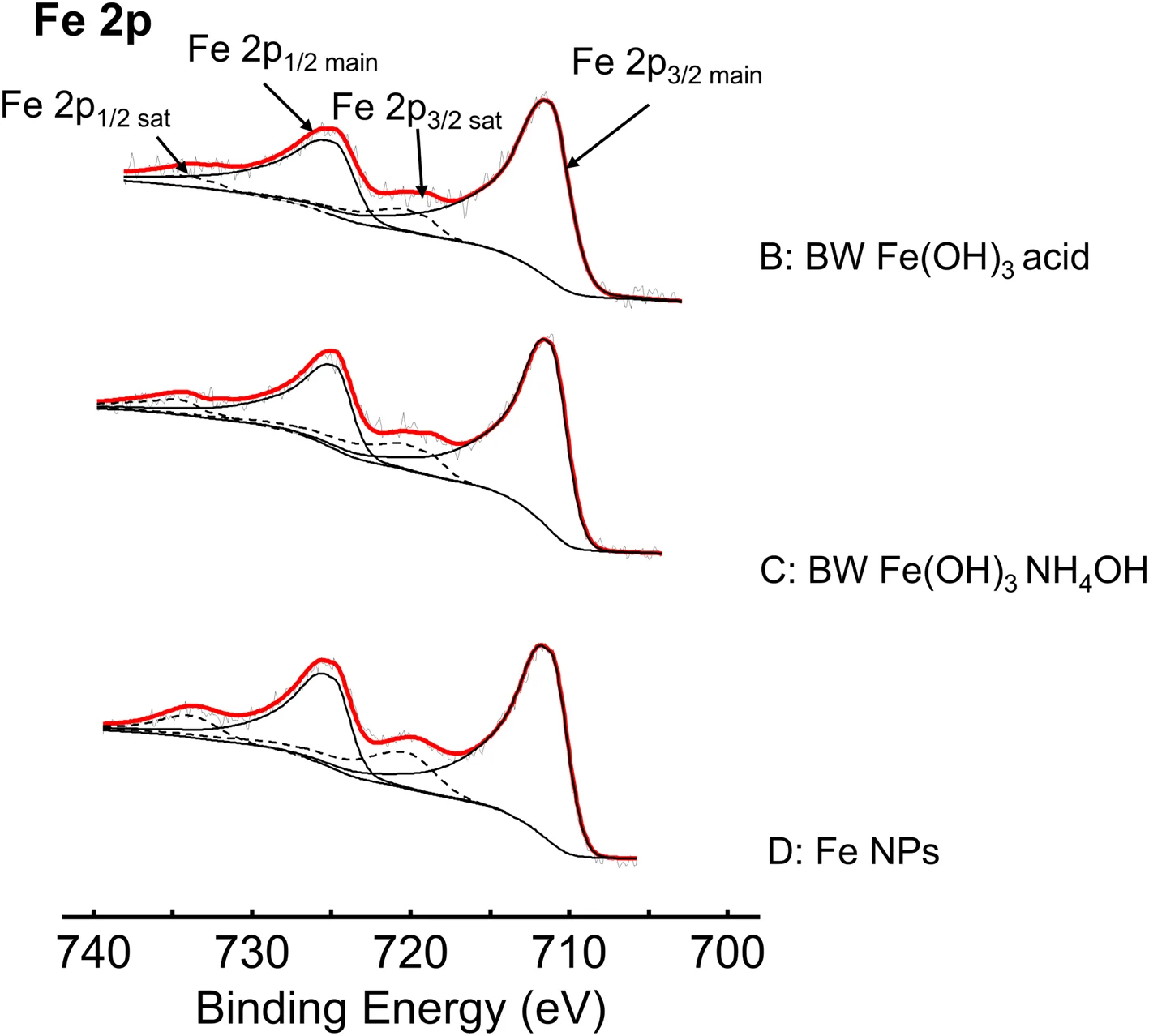
High-resolution Fe 2p spectra showing main Fe 2p_3/2_ peaks at 710.8–711.2 eV and characteristic Fe^3+^ satellites at 719–720 eV (arrows), confirming Fe_2_O_3_/FeOOH phases.

High-resolution Fe 2p spectra (Fig. 5) were used to evaluate the oxidation state of iron in the modified samples. All iron-containing samples exhibit a prominent Fe 2p_3/2_ peak at 710.8–711.2 eV together with characteristic satellite features located between 719.0 and 720.0 eV. The measured spin–orbit splitting (ΔBE = 13.6 eV) between the Fe 2p_3/2_ and Fe 2p_1/2_ (∼724.7 eV) peaks is characteristic of Fe(iii)-containing species and are consistent with the presence of iron oxide and/or iron oxyhydroxide phases (Table 2).

**Table 2:** The XPS binding energies (eV) and *i*_ratio_ values of the main and satellite Fe 2p peaks

Sample	Fe_main_ (eV)		Fe_sat_ (eV)		ΔBE_Fe 2p1/2main − Fe 2p3/2main_ (eV)	*i*_ratio_ = (*i*_sat_/(*i*_sat_ + *i*_main_))
	2p_3/2_	2p_1/2_	2p_3/2_	2p_1/2_		
B	711.2	724.7	719.2	732.5	13.5	0.14
C	711.2	724.7	719.2	732.5	13.5	0.13
D	711.1	724.7	719.0	732.5	13.6	0.18

Furthermore, the absence of a pronounced feature near 709.5 eV suggests that Fe^2+^ species are either absent or present only in minor quantities at the surface. The calculated satellite-to-main peak intensity ratios (*i*_ratio_) were 0.14 for Sample B, 0.13 for Sample C, and 0.18 for Sample D (Table 2). The Fe 2p spectral features are consistent with predominantly Fe(iii)-containing surface species. The satellite-to-main peak intensity ratios are not interpreted as quantitative measures of total Fe concentration, particle size, or active-site density. The present XPS data provide surface-chemical evidence for Fe-associated phases but do not establish metallic Fe, zero-valent iron, or nanoscale particle dimensions.

XPS provides surface-sensitive evidence that Samples B–D contain Fe-associated species with predominantly Fe(iii) character. The C 1s and O 1s component assignments are interpreted as semi-quantitative fitted peak-area contributions and are not used as measurements of bulk composition, total Fe loading, particle size, or active-site density. For Sample D, the Fe 2p signal supports Fe(iii)-containing surface species, whereas the O 1s fitting does not resolve a distinct Fe–OH component. Therefore, the XPS results are used to support qualitative surface-chemical differences among the materials rather than to establish a unique Fe coordination environment or molecular adsorption mechanism.^24–26^ The present XPS and SEM-EDS results provide surface-sensitive and local evidence for Fe-associated phases but do not determine total Fe loading, particle-size distribution, or the distribution of Fe throughout the bulk adsorbent. Therefore, adsorption-performance differences among Samples B–D cannot be assigned quantitatively to total Fe content or nanoscale particle properties. A limitation of the present study is that total bulk Fe was not quantified by ICP-OES, ICP-MS, or XRF. Consequently, the relationship between nominal iron formulation, total Fe content, and adsorption performance cannot be quantified, and the discussion is restricted to the measured surface-characterisation results.

### 3.4 Analytical validation and final solution pH

The UV-vis calibration curves obtained at nominal pH 3–11 were linear over the tested concentration range, with *R*^2^ values of 0.9988–0.9991 (Table S6, SI). Membrane-filter recoveries ranged from 98.4 to 99.6%, while matrix spike recoveries ranged from 96.8 to 101.2% (Table S7, SI). The adsorbent-blank absorbance at 357 nm was low, ranging from 0.002 to 0.005. The final pH values of the TC adsorption solutions differed from their initial values by −0.28 to +0.32 pH units across Samples A–D (Table S8, SI). These results indicate that solution pH was broadly maintained during the pH-dependent adsorption experiments. Chromatographic confirmation by HPLC was not available in the present study; therefore, the UV-vis method was supported through pH-specific calibration, membrane-filter recovery, matrix spike-recovery, blank assessment, and reporting of final solution pH.

#### 3.4.1 Point of zero charge and isoelectric point

**Fig. 6:**
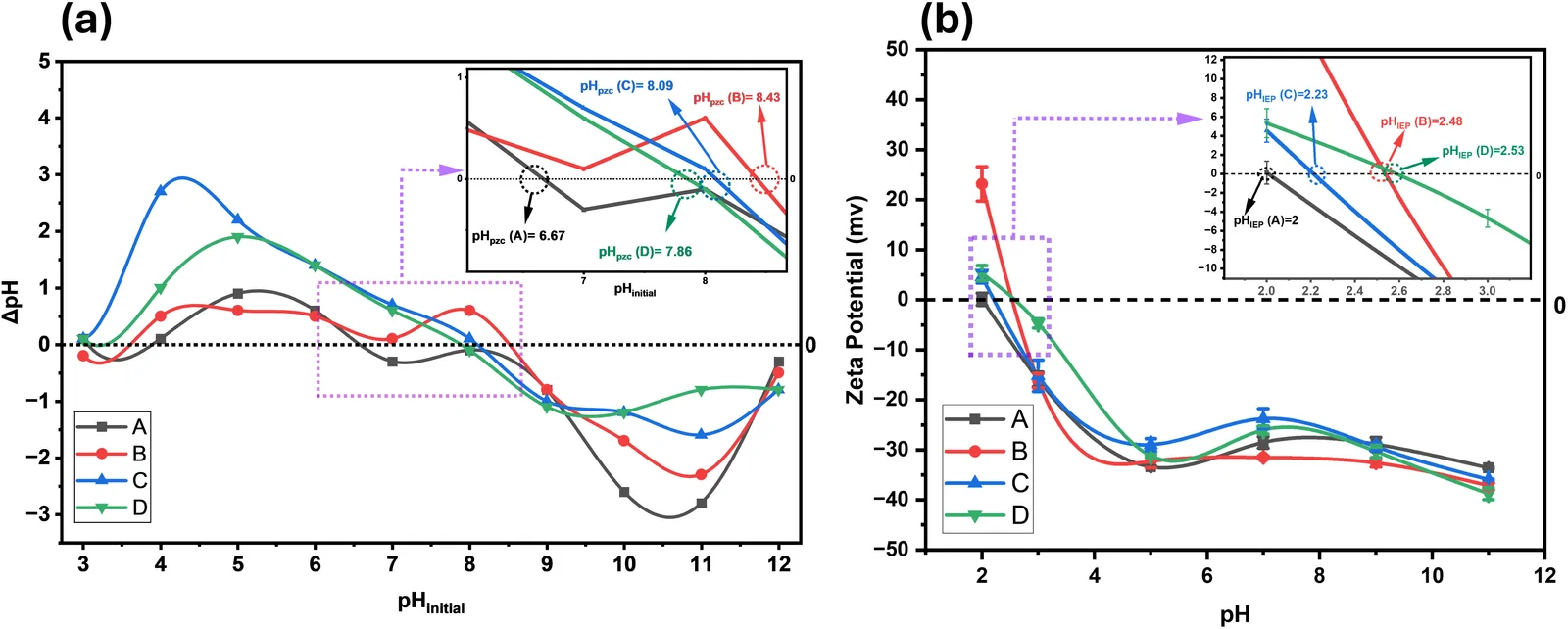
Surface-charge characterisation of Samples A–D: (a) pH-drift curves used to estimate the operational pH_pzc_, where ΔpH = pH_f_ − pH_i_; and (b) zeta potential as a function of pH, used to estimate pH_IEP_. The pH_pzc_ and pH_IEP_ values represent different interfacial properties and are not used interchangeably to assign bulk electrostatic attraction or repulsion.

The point of zero charge (*c*) values of Samples A–D were determined by the pH-drift method, and the isoelectric points (IEP) were obtained from zeta-potential measurements as a function of pH (Fig. 6a and b). Sample A exhibited pH_pzc_ = 6.67, whereas the Fe-modified materials B–D showed higher pH_pzc_ values of 8.43, 8.09 and 7.86, respectively, reflecting the contribution of surface Fe(iii) oxyhydroxide groups. The IEP values were 2.00, 2.48, 2.23 and 2.53 for Samples A–D, respectively, indicating that all sorbents carry a net positive zeta potential below pH ≈ 2–2.5 and become increasingly negative at higher pH.

The pH_pzc_ and pH_IEP_ values provide complementary but distinct information about the adsorbent–solution interface. The pH_pzc_, determined by the pH-drift method, represents the pH at which the net proton charge of the total adsorbent surface is zero. In contrast, pH_IEP_, determined from zeta-potential measurements, represents the pH at which the electrokinetic potential at the hydrodynamic shear plane is zero. The separation between pH_pzc_ and pH_IEP_ indicates that these quantities should not be used interchangeably.

At the adsorption-relevant pH values of 5 and 7, the measured zeta potentials were negative for all adsorbents. Therefore, bulk electrostatic attraction between TC and the adsorbent surface cannot be inferred solely from the pH_pzc_. The pH_pzc_ and pH_IEP_ values provide complementary but distinct information about the adsorbent–solution interface. The pH_pzc,_ determined by the pH-drift method, represents the pH at which the net proton charge of the total adsorbent surface is zero. In contrast, pH_IEP_, determined from zeta-potential measurements, represents the pH at which the electrokinetic potential at the hydrodynamic shear plane is zero. The separation between pH_pzc_ and pH_IEP_ indicates that these quantities should not be used interchangeably.

At the adsorption-relevant pH values of 5 and 7, the measured zeta potentials were negative for all adsorbents. Therefore, bulk electrostatic attraction between TC and the adsorbent surface cannot be inferred solely from the pH_pzc_ values. The pH-dependent adsorption behaviour is interpreted cautiously in terms of combined effects of TC speciation, electrokinetic behaviour, carbonaceous surface functionality, pore accessibility, and possible interactions with Fe-associated surface species.

TC exists predominantly as a cationic species below pH 3.3, as a zwitterionic species between approximately pH 3.3 and 7.7, and as increasingly anionic species above pH 7.7. Sample A showed maximum removal at pH 7, while Samples B and C showed maximum removal at pH 5, and Sample D showed maximum removal at pH 7. These trends cannot be explained using a simple positively charged-surface/negatively charged-solute model. The present data do not directly establish the local charge of Fe-associated surface sites or a unique molecular adsorption mechanism.

The pH_pzc_ values are therefore treated as operational estimates from the pH-drift method, whereas the zeta-potential data provide the direct experimental indication of electrokinetic surface behaviour.

TC exists predominantly as a cationic species below pH 3.3, as a zwitterionic species between approximately pH 3.3 and 7.7, and as increasingly anionic species above pH 7.7. Sample A showed maximum removal at pH 7, while Samples B and C showed maximum removal at pH 5, and Sample D showed maximum removal at pH 7. These trends cannot be explained using a simple positively charged-surface/negatively charged-solute model. The present data do not directly establish the local charge of Fe-associated surface sites or a unique molecular adsorption mechanism. The pH_pzc_ values are therefore treated as operational estimates from the pH-drift method, whereas the zeta-potential data provide the direct experimental indication of electrokinetic surface behaviour.

### 3.5 Effect of adsorbent dosage on tetracycline removal

**Fig. 7:**
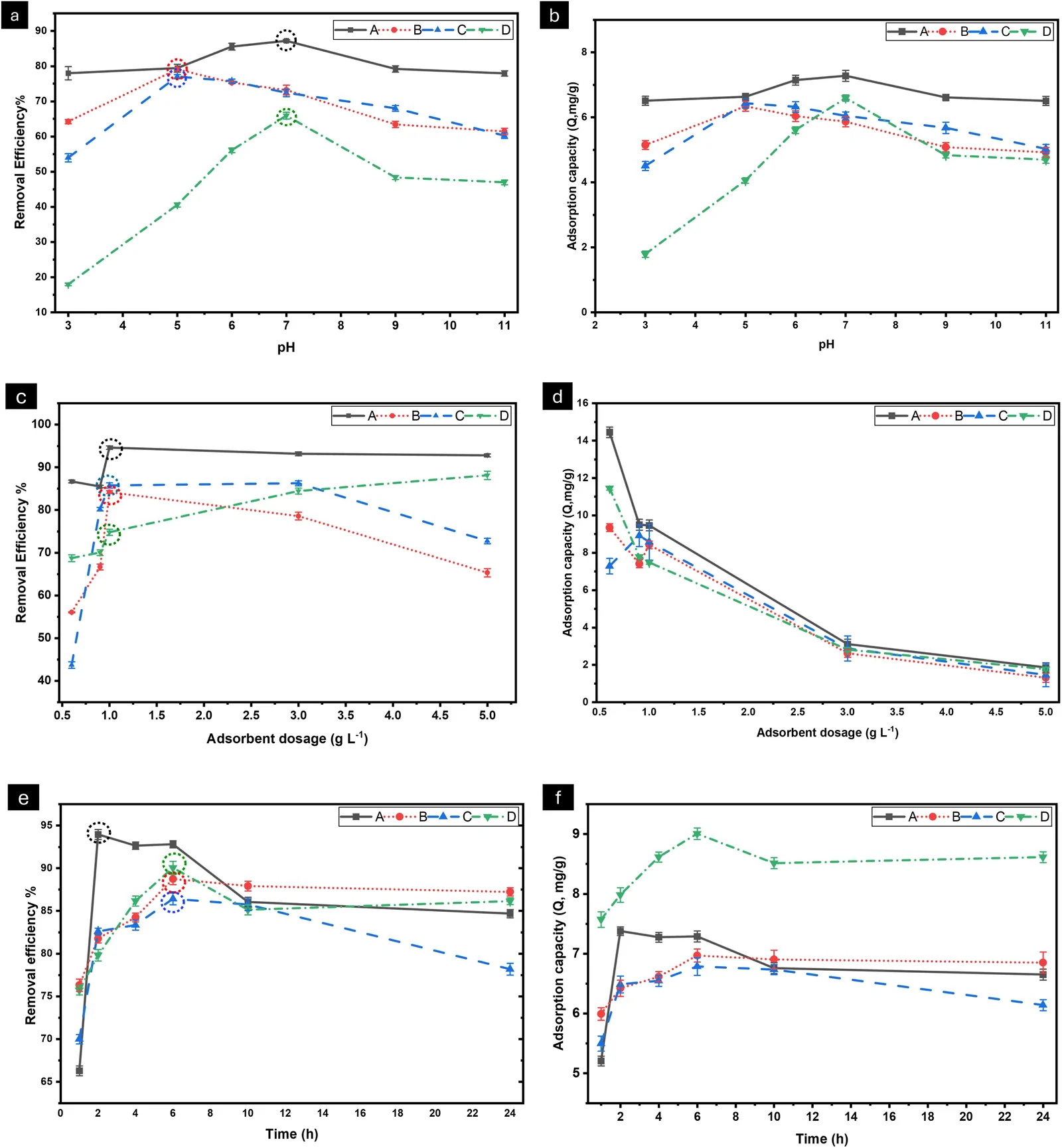
Effect of (a and b) solution pH, (c and d) adsorbent dosage, and (e and f) contact time on tetracycline adsorption under optimised conditions. (a) Removal efficiency (*R*%) *vs.* pH for sorbents A–D at *C*_0_ = 10 mg L^−1^, dosage 1.0 g L^−1^, *V* = 0.010 L. (b) Corresponding equilibrium capacity *q*_e_ (mg g^−1^) *vs.* pH from *q*_e_ = (*C*_0_ − *C*_e_)*V/m*. (c) *R*% *vs.* adsorbent dosage (0.6–5.0 g L^−1^) at *C*_0_ = 10 mg L^−1^, optimised pH for each sorbent. (d) *q*_e_*vs.* dosage, recalculated with *q*_e_ = (*C*_0_ − *C*_e_)*V*/*m* using *V* = 0.010 L and *m* = dosage × *V* to ensure mass-balance consistency. (e and f) Time-dependent removal and capacity profiles at *C*_0_ = 10 mg L^−1^ and dosage 1.0 g L^−1^.

Fig. 7c and d illustrate the influence of adsorbent dosage on TC removal efficiency (*R*%) and equilibrium capacity (*q*_e_), evaluated across a concentration range of 0.6 to 5.0 g L^−1^ under optimized matrix pH levels (pH 7.0 for Samples A and D; pH 5.0 for Samples B and C) at 25 °C for an initial screening window of 120 min. Sample A reached a high baseline removal efficiency of 94.6 ± 0.7% with a corresponding adsorption capacity of 8.4 ± 0.9 mg g^−1^ at an optimal dosage of 1.0 g L^−1^. At this identical dosage parameter, Sample B achieved its maximum localized clearance performance of 84.2 ± 0.3% (*q*_e_ = 7.5 ± 1.0 mg g^−1^), while Sample C demonstrated 85.7 ± 0.6% removal efficiency (*q*_e_ = 7.6 mg g^−1^). In contrast, Sample D achieved its maximum total antibiotic removal profile (88.1 ± 1.0%) at the highest evaluated loading boundary of 5.0 g L^−1^. However, tracking the capacity vectors revealed that its unit adsorption performance decreased from 11.45 ± 0.89 mg g^−1^ at a dosage of 0.6 g L^−1^ down to 7.48 ± 1.03 mg g^−1^ at 1.0 g L^−1^, eventually stabilizing at 1.76 ± 0.10 mg g^−1^ at 5.0 g L^−1^.

These combined profiles demonstrate a clear inverse relationship between the dry mass loading of the functional sorbent and its specific unit adsorption capacity (*q*_e_), a trend common to both raw and mineral-functionalized bio-based frameworks. As the solid dosage increases, the cumulative number of active surface configurations and exposed anchoring sites scales up, increasing fluid boundary collision probabilities and maximising free surface areas available to trap ambient TC species.^27^ Conversely, when working with a fixed initial antibiotic concentration (*C*_0_ = 10 mg L^−1^), elevating the solid-to-liquid ratio yields a higher percentage of underutilised, vacant, and chemically unsaturated binding sites at equilibrium, which mathematically lowers the calculated unit capacity. This behaviour is also driven by severe fluid mass transfer limitations. As the bulk concentration of liquid-phase TC drops rapidly at high solid loadings, the concentration gradient driving force decreases, and localised particle aggregation or screening effects occur at elevated solid boundaries, reducing the net accessible surface area per unit mass.^28^

Sorbent A (*Acacia mearnsii* iron-free biochar) displayed high clear-zone efficiency, reaching 94.6 ± 0.7% removal at 1.0 g L^−1^ (*q*_e_ = 8.4 ± 0.9 mg g^−1^). While its capture performance was already strong at a low dosage of 0.6 g L^−1^ (86.7 ± 0.7%), it expanded by 7.9% when moving up to the 1.0 g L^−1^ threshold. Further increases in solid loading to 3.0 and 5.0 g L^−1^ provided no measurable benefits, causing minor efficiency declines to 93.1% and 92.8%, respectively. This stagnation is driven by physical particle agglomeration, which blinds active surface structures and blocks accessible boundary layers. Consequently, the unit capacity of sorbent A fell steeply from 12.9 ± 0.9 mg g^−1^ at 0.6 g L^−1^ down to 1.6 ± 0.6 mg g^−1^ at 5.0 g L^−1^ (Fig. 7d), confirming active site saturation at high solid-to-liquid ratios. This behaviour aligns with literature benchmarks for raw biomass biochar, where optimal processing dosages typically fall between 0.5 and 2.0 g L^−1^ to balance material economy with target decontamination goals.^29^

Sample B (*Acacia mearnsii* matrix loaded with 25 wt% Fe(OH)_3_ under acidic precipitation) exhibited its highest removal efficiency at a precise threshold of 1.0 g L^−1^ (84.2 ± 0.3%, *q*_e_ = 7.5 ± 1.0 mg g^−1^), showing weaker relative retention parameters at both lower (56.0% at 0.6 g L^−1^) and higher (78.6% at 3.0 g L^−1^; 65.3% at 5.0 g L^−1^) solid settings. The decrease in removal efficiency at high adsorbent dosage may reflect reduced effective site utilisation, particle aggregation, changes in the concentration driving force, or mass-transfer limitations. The present data do not distinguish among these contributions.^30^ The notable 28.2% jump in performance when expanding the dose from 0.6 to 1.0 g L^−1^ confirms that TC capture is restricted by site limitations at sub-optimal solid fractions. The subsequent drop at 5.0 g L^−1^ indicates that extreme particle crowding restricts access to the surface iron coordination sites and alters local solution chemistry *via* high solid loading.

Sample C (*Acacia mearnsii* matrix loaded with 25 wt% Fe(OH)_3_ under NH_4_OH stabilisation) showed an efficiency jump from 43.7 ± 0.8% at 0.6 g L^−1^ up to 85.7 ± 0.6% at 1.0 g L^−1^, plateauing at 86.3 ± 0.6% at 3.0 g L^−1^. The restricted performance at 0.6 g L^−1^ highlights poor active site accessibility at low dosages, while the similar performance at 1.0 and 3.0 g L^−1^ shows that a 1.0 g L^−1^ threshold provides enough operational sites to reach equilibrium. The downward trend observed at 5.0 g L^−1^ mirrors. Sample B, driven by mass transfer constraints and mineral agglomeration. Its calculated unit capacity fell from 14.6 mg g^−1^ at 0.6 g L^−1^ to 1.3 ± 0.6 mg g^−1^ at 5.0 g L^−1^, confirming that high-dosage parameters are economically inefficient.

Unlike the wattle-derived materials, Sample D displayed a continuous increase in antibiotic removal efficiency across the entire evaluated dosage window, rising from 68.7 ± 0.8% at 0.6 g L^−1^ to 88.1 ± 1.0% at 5.0 g L^−1^. This indicates that its active surface sites remain uncompromised by saturation at 5.0 g L^−1^, or that its unique fibre geometry enhances fluid mass transfer dynamics *via* high localised particle dispersion. However, this configuration caused a major drop in specific capacity, which fell by 84.6% from 11.45 ± 0.89 mg g^−1^ at 0.6 g L^−1^ down to 1.76 ± 0.10 mg g^−1^ at 5.0 g L^−1^.

This high low-dosage capacity stems from the high density, uniform dispersion, and superior chemical accessibility of the iron oxide/oxyhydroxide-carbon composite coordination sites across the open fibre micro-scaffold. Elevating the solid dose leaves a large fraction of these active metal centres underutilised. Engineered iron nanoparticles often display similar dose-dependent properties: lower solid thresholds (<1 g L^−1^) maximise unit utilisation capacity, whereas high concentrations (>3 g L^−1^) improve total removal percentages but decrease overall material efficiency.

While Samples A, B, and C show optimal performance balance at a fixed threshold of 1.0 g L^−1^, Sample D exhibits distinct advantages, maintaining an operational capacity of 7.48 mg g^−1^ at this same 1.0 g L^−1^ marker. For scale-up designs, selecting an optimised processing dose requires balancing removal efficiency against engineering economy. Although a 3.0 g L^−1^ dose of Sample D offers a higher total removal percentage than a 1.0 g L^−1^ dose (84.4% *vs.* 74.8% at 120 min), the 1.0 g L^−1^ threshold is recommended for large-scale operations. This setting optimises material efficiency (7.48 mg g^−1^*vs.* 2.81 mg g^−1^), minimises total operational costs, and delivers sufficient target clearance.

The observed low-dosage performance of Sample D may reflect differences in fibre morphology, accessible surface area, and surface chemistry relative to the other materials; however, the present data do not isolate the contribution of individual structural or chemical features. This makes the fibre-templated material highly suitable for multi-stage treatment designs or cyclical regeneration operations. Sample A utilized its native *Acacia*-derived proanthocyanidin chemistry to drive multiple non-covalent binding pathways, achieving a high removal efficiency (94.6%) at 1.0 g L^−1^. While Sample D provided the highest low-dosage capacity among the Fe-modified materials (11.45 mg g^−1^ at 0.6 g L^−1^), while Sample A showed the highest absolute capacity at 0.6 g L^−1^ (12.86 mg g^−1^), it required larger solid masses to match the total clearance percentages of Sample A. Samples B and C showed intermediate performance because their bulk iron networks were more clustered and less accessible than the open nanoparticle arrays on Sample D. Balancing these factors, a standardized dosage of 1.0 g L^−1^ was selected across all four materials for subsequent kinetic and equilibrium isotherm modelling.

### 3.6 Effect of contact time on tetracycline removal

The influence of contact time on TC removal metrics was evaluated under optimised matrix pH and solid dosage parameters (1.0 g L^−1^), tracking both total removal percentage (*R*%) and dynamic uptake capacity (*q*_*t*_) across an operating window from 20 min to 24 h (Fig. 7e and f). All four matrices displayed a rapid initial adsorption rate during the first few hours of fluid contact, followed by a slower progression toward equilibrium. Sample A exhibited the fastest initial uptake, reaching 66.29% removal at 1 h and 93.95% at 2 h, and maintaining a stable operational plateau thereafter (92.6–92.8%). This trend indicates that readily accessible adsorption sites contributed substantially to the initial uptake of TC by Sample A. The specific contributions of aromatic interactions and hydrogen bonding are considered plausible from the carbon surface chemistry but cannot be quantified from contact-time data alone. This initial uptake profile indicates that readily accessible adsorption sites contributed to TC removal during the early contact period. The present contact-time data do not independently identify the molecular nature of the surface interaction.^31^ Interestingly, extending contact times further (10–24 h) caused minor declines in both *R*% and *q*_*t*_ for Sample A, confirming the reversible nature of these weaker physisorptive interactions.

For Sample B, equilibrium was reached more gradually; its removal efficiency increased from 76.3% at 1 h to a maximum of 88.7% at 6 h, stabilizing at a dynamic capacity of 8.87 mg g^−1^. Both removal and capacity values remained constant between 6 and 24 h, showing that true equilibrium was achieved within this window. The gradual approach to a plateau for Sample B indicates that uptake continued beyond the initial adsorption period. This behaviour is consistent with a combination of external mass transfer, adsorption at accessible sites, and slower transport within the porous structure; it does not alone establish a chemisorption or inner-sphere complexation mechanism.

Sample C followed a similar kinetic trend, with total removal rising from 69.9% at 1 h to 84.6% at 6 h, corresponding to an equilibrium capacity of 8.46 mg g^−1^, before reaching full stability at 10 h. However, unlike Sample B, Sample C exhibited a small decline in capacity parameters at 24 h. The minor decrease observed after prolonged contact may reflect experimental variability, reversible adsorption, or changes in solution–surface equilibrium. The present data do not distinguish among these possibilities. This behaviour indicates a gradual approach to an apparent plateau under the tested conditions. The contact-time data alone do not distinguish among surface interaction, diffusion, and solution–surface equilibrium contributions.

Fig. 7e and f illustrate that Sample D displays rapid initial uptake kinetics, with its removal efficiency rising from 75.7% to 90.0% within the first 6 h, achieving a dynamic capacity of 9.01 ± 0.17 mg g^−1^. Its high capacity remained stable across the 10 h and 24 h tracking markers (retaining *R*% ≈ 85.1–86.1), indicating that the measured TC-removal performance remained broadly similar between the 6 and 24 h sampling points under the tested laboratory conditions. This kinetic behaviour matches literature profiles for engineered nanoscale iron composites, which typically reach equilibrium within 4–8 h and maintain stable long-term retention.

All four materials completed at least 80% of their total target antibiotic capture within the first 2 to 6 h of contact, indicating a highly responsive initial adsorption stage typical of porous carbon substrates with strong sorbent–sorbate affinity. The clear plateau regions observed for Samples A, B, and D between 4 and 10 h show consistent secondary capture rates that match literature trends for iron–carbon hybrid materials.^32^ Samples A and C showed minor changes in removal performance over the extended contact-time experiment. The available data do not permit assignment of these changes to a specific adsorption mechanism.^33,34^ For subsequent laboratory adsorption experiments, contact times of 2–6 h for A and D and 6–10 h for B and C were selected on the basis of the observed uptake profiles. These times should be regarded as laboratory equilibrium conditions rather than design values for a full-scale treatment process.

**Fig. 8:**
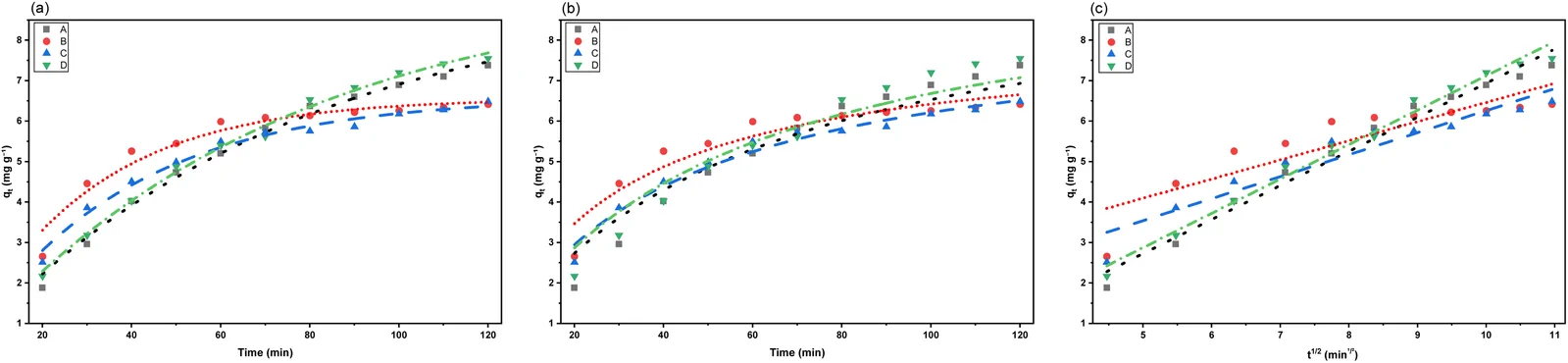
Non-linear kinetic analysis of TC adsorption onto Samples A–D: (a) pseudo-first-order model, (b) pseudo-second-order model, and (c) Weber–Morris intraparticle-diffusion plot. Symbols represent experimental *q*_*t*_ data collected from 20 to 120 min, and lines represent fitted model curves. Kinetic fitting used the 20–120 min dataset only. Complete regression parameters are reported in Tables 3 and S5, SI.

The extended 20 min–24 h contact-time experiment was used to identify practical equilibration periods for batch optimisation, whereas the kinetic model parameters reported in Section 3.7 were calculated from the separate, higher-resolution 20–120 min dataset shown in Fig. 8.

### 3.7 Kinetic modelling of TC adsorption

The adsorption kinetics of tetracycline (TC) onto Samples A–D were evaluated using non-linear pseudo-first-order (PFO) and pseudo-second-order (PSO) equations fitted directly to the experimental *q*_*t*_-*versus*-time data collected between 20 and 120 min (Fig. 8a and b). The PFO model was expressed as (eqn (4)):

4 *q*_*t*_ = *q*_e_(1 − *e*^−*k*_1_*t*^)

where *q*_*t*_ and *q*_e_ are the TC uptake capacities at time and equilibrium, respectively, and is the PFO rate constant. The PSO model was expressed as (eqn (5)):5
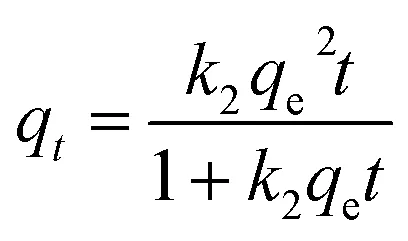
where *k*_2_ is the PSO rate constant. Non-linear regression was used because fitting transformed linear equations can alter the distribution of experimental error and generate biased kinetic parameters.^35,36^

The PFO model provided a better statistical description of the kinetic data for all four samples. The PFO *R*^2^ values were 0.992, 0.950, 0.986, and 0.995 for Samples A, B, C, and D, respectively, compared with PSO *R*^2^ values of 0.909 and 0.975 for Samples B and C. The non-linear PFO equilibrium capacities were 9.22 ± 0.37 mg g^−1^ for A, 6.57 ± 0.16 mg g^−1^ for B, 6.61 ± 0.12 mg g^−1^ for C, and 9.48 ± 0.31 mg g^−1^ for D. These fitted values are physically consistent with the batch mass-balance limit of 10 mg g^−1^ under the kinetic conditions (*C*_0_ = 10 mg L^−1^, *V* = 0.010 L, and *m* = 0.010 g). Complete non-linear PFO and PSO fitting parameters, including parameter uncertainty, adjusted *R*^2^, residual sum of squares, reduced *χ*^2^, and fit status, are provided in Table S5, SI.

For Samples A and D, unconstrained PSO fitting produced equilibrium capacities exceeding the total amount of TC initially present in the batch system. Therefore, PSO fitting was constrained at *q*_e_ = 10 mg g^−1^, but the constrained regressions did not converge successfully and gave lower *R*^2^ values than PFO. These constrained PSO results are provided only for completeness and were not used to identify the best kinetic model. For Samples B and C, the PSO model yielded fitted equilibrium capacities of 8.15 ± 0.48 and 8.59 ± 0.34 mg g^−1^, respectively; however, the PFO model still provided superior fits based on *R*^2^, adjusted *R*^2^, and residual error.

The PFO rate constants followed the order B > C > A ≈ D, with *k*_1_ values of 0.0350, 0.0277, 0.0139, and 0.0139 min^−1^ for B, C, A, and D, respectively. Thus, Sample B showed the fastest approach to its fitted equilibrium capacity, whereas Samples A and D displayed higher fitted uptake capacities under the fixed low-concentration conditions. These results describe the macroscopic uptake rate only. They do not independently demonstrate physisorption, chemisorption, or inner-sphere complexation, because PFO and PSO models are empirical rate equations rather than molecular-scale mechanistic probes. Specific interactions between TC functional groups and Fe(iii) oxide/oxyhydroxide sites should therefore be discussed as a plausible contribution supported by the Fe 2p and O 1s XPS results, rather than as a conclusion derived from kinetic fitting alone.^36^

The Weber–Morris intraparticle-diffusion model was applied according to *q*_*t*_ = *k*_id_*t*^1/2^ + *C*, where *k*_id_ is the intraparticle-diffusion rate constant and *C* represents the boundary-layer contribution. The full-range IPD plots did not pass through the origin. Samples B and C gave positive intercepts of 1.730 and 0.826 mg g^−1^, respectively, indicating a measurable external-film or boundary-layer contribution. Samples A and D gave negative intercepts when one linear equation was fitted across the complete 20–120 min dataset. These negative values are not physically interpretable as boundary-layer thicknesses; instead, they indicate that one linear IPD equation does not adequately represent the complete uptake profile. Accordingly, the present full-range IPD analysis supports the conclusion that intraparticle diffusion contributes to TC uptake but is not the sole rate-limiting step. It does not justify assigning a unique diffusion-controlled mechanism without statistically justified multi-stage fitting.

The PFO model was preferred for all samples, while IPD was contributory but not solely rate-controlling. The observed adsorption behaviour is consistent with simultaneous contributions from external mass transfer, adsorption at accessible carbonaceous and Fe-containing surface sites, and diffusion into the pore structure. The relative importance of electrostatic interactions, hydrogen bonding, aromatic interactions, and Fe-associated complexation depends on solution pH, TC speciation, and the surface chemistry of each material.

The kinetic profiles showed a progressive increase in TC uptake over 20–120 min, with distinct adsorption-rate behaviour among the samples. Sample B displayed the fastest approach to its fitted equilibrium capacity, as reflected by its comparatively high PFO rate constant (*k*_1_ = 0.0350 min^−1^), whereas Samples A and D exhibited higher fitted capacities but continued to increase in uptake at the final measured time point. Under the present experimental window, the PFO model provided the most statistically appropriate empirical description of the uptake profiles for all samples.

The kinetic results demonstrate that the uptake profiles of Samples A–D are more accurately described by the non-linear PFO model than by the PSO model under the present low-concentration batch conditions. Sample B had the highest apparent rate constant, whereas Samples A and D showed the highest fitted capacities without exceeding the physical mass-balance limit. The full-range Weber–Morris plots did not support intraparticle diffusion as the sole rate-controlling mechanism. TC adsorption is therefore interpreted as a combined process involving mass transfer from solution, interaction with accessible carbonaceous and Fe-containing surface sites, and diffusion into the pore structure. The molecular contribution of aromatic interactions, hydrogen bonding, electrostatic effects, and Fe-associated complexation should be evaluated together with the pH-dependent adsorption, XPS, and FTIR results, rather than being inferred from kinetic model selection alone (Table 3).

**Table 3:** Preferred non-linear PFO kinetic parameters and Weber–Morris intraparticle-diffusion parameters for TC adsorption onto Samples A–D

Sample	PFO *q*_e_ (mg g^−1^)	PFO *k*_1_ (min^−1^)	PFO *R*_2_	*k*_id_ (mg g^−1^ min^−1/2^)	*C* (mg g^−1^)	*R*_IPD_^2^
A	9.22 ± 0.37	0.0139 ± 0.0010	0.992	0.843 ± 0.042	−1.480 ± 0.348	0.979
B	6.57 ± 0.16	0.0350 ± 0.0031	0.950	0.473 ± 0.084	1.730 ± 0.701	0.780
C	6.61 ± 0.12	0.0277 ± 0.0015	0.986	0.544 ± 0.055	0.826 ± 0.461	0.915
D	9.48 ± 0.31	0.0139 ± 0.0008	0.995	0.850 ± 0.034	−1.372 ± 0.286	0.986

PFO parameters were obtained by non-linear regression of untransformed *q*_*t*_-*versus*-time data. Weber–Morris parameters were obtained from full-range linear fits of *q*_*t*_*versus t*^1/2^ over 20–120 min. The negative *C* values for Samples A and D indicate that a single full-range IPD line does not provide a physically meaningful boundary-layer thickness for these samples; therefore, IPD is interpreted as contributory rather than as the sole rate-controlling process.

### 3.8 Adsorption isotherms and surface energetics

**Fig. 9:**
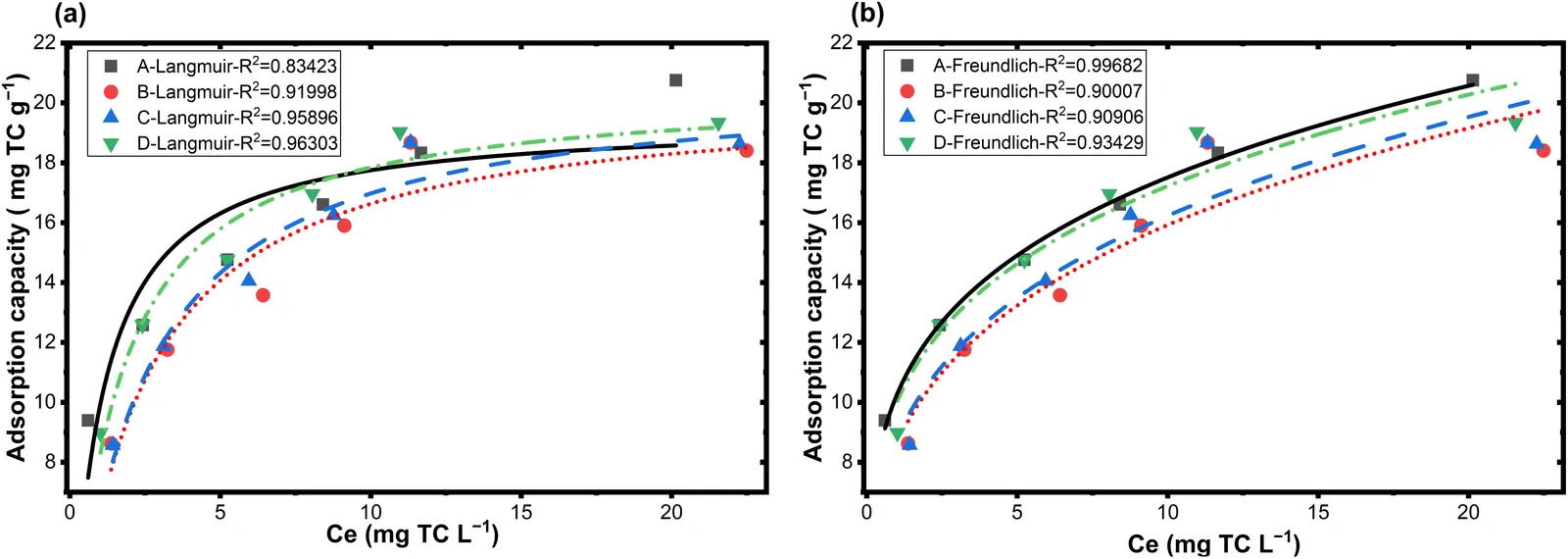
Non-linear equilibrium isotherm fits for tetracycline (TC) adsorption onto Samples A–D. (a) Langmuir model and (b) Freundlich model showing adsorption capacity *q*_e_ as a function of equilibrium concentration *C*_e_ at 25 °C. Symbols represent experimental data and lines represent non-linear regression fits for each sorbent; the corresponding *R*^2^ values are given in the legends.

The equilibrium adsorption data were first analysed using the Langmuir and Freundlich isotherm models. Non-linear regression of the original Langmuir and Freundlich equations is presented in Fig. 9, and the corresponding parameters are summarised in Table 4. The linearised Langmuir plots (*C*_e_/*q*_e_*versus C*_e_) and Freundlich plots (ln *q*_e_*versus* ln *C*_e_) are shown for comparison in Fig. S6 (SI). For Sample A, the nonlinear Freundlich model provided the best description of the equilibrium data, with *K*_F_ = 10.3 ± 0.2, *n* = 4.31 ± 0.14, adjusted *R*^2^ = 0.996, reduced *χ*^2^ = 0.066, and SSE = 0.264 (Tables 4 and S4).

**Table 4:** Preferred nonlinear isotherm models and fitted parameters for tetracycline adsorption on Samples A–D^a^

Sample	Preferred nonlinear model	Parameter 1	Parameter 2	Adj. *R*^2^	Reduced *χ*^2^	SSE
A	Freundlich	*K*_F_ = 10.3 ± 0.2	*n* = 4.31 ± 0.14	0.996	0.066	0.264
B	Langmuir	*q*_m_ = 20.3 ± 1.4 mg g^−1^	*K*_L_ = 0.449 ± 0.120 L mg^−1^	0.900	1.55	6.20
C	Langmuir	*q*_m_ = 20.8 ± 1.0 mg g^−1^	*K*_L_ = 0.439 ± 0.082 L mg^−1^	0.949	0.817	3.27
D	Langmuir	*q*_m_ = 20.5 ± 0.8 mg g^−1^	*K*_L_ = 0.671 ± 0.116 L mg^−1^	0.954	0.745	2.98

a The Langmuir *q*_m_ values are model-derived parameters obtained from equilibrium isotherm experiments conducted at *C*_0_ = 10–40 mg L^−1^, *V* = 10.0 mL, and adsorbent mass = 0.010 g. Under these conditions, the concentration-dependent batch mass-balance maximum ranged from 10 to 40 mg g^−1^. The 10 mg g^−1^ mass-balance limit applies only to the separate kinetic and optimisation experiments performed at *C*_0_ = 10 mg L^−1^.

The high *n* value and 1/*n* < 1 indicate favourable adsorption on an energetically heterogeneous surface. This behaviour is consistent with the chemically diverse *Acacia mearnsii*-derived carbon matrix, where TC adsorption is likely governed by a combination of π–π interactions, hydrogen bonding, and interactions with oxygen-containing surface groups. Similar Freundlich-dominated adsorption behaviour has been reported for tetracycline adsorption on heterogeneous soil and carbonaceous materials.^37,38^

In contrast, Samples B, C, and D were better described by the non-linear Langmuir model. The fitted Langmuir capacities were 20.33 ± 1.35, 20.85 ± 1.00, and 20.51 ± 0.79 mg g^−1^ for B, C, and D, respectively. The corresponding Langmuir affinity constants were 0.449 ± 0.120, 0.439 ± 0.082, and 0.671 ± 0.116 L mg^−1^. These parameters were obtained from the isotherm dataset collected across initial concentrations of 10–40 mg L^−1^ and must not be interpreted as experimentally achieved capacities under the separate fixed-concentration kinetic condition of *C*_0_ = 10 mg L^−1^. The stronger Langmuir fit for these iron-containing adsorbents suggests adsorption onto more defined surface sites, most likely associated with Fe-containing phases. The higher *K*_L_ value of Sample D indicates stronger apparent adsorbent–adsorbate affinity, which can be attributed to the improved accessibility and dispersion of surface iron oxide/oxyhydroxide phases on the cellulose-derived carbon fibre support. Similar Langmuir-type behaviour has been reported for metal-modified biochar and iron-based adsorbents where adsorption occurs predominantly on relatively uniform active sites.^39–42^

The dimensionless separation factor values (*R*_L_ = 0.16–0.23 at *C*_0_ = 10 mg L^−1^) fall within the favourable adsorption range of 0 < *R*_L_ < 1 for all adsorbents, confirming that TC adsorption is favourable under the experimental conditions. The lower *R*_L_ value for Sample D further supports its stronger apparent affinity toward TC, consistent with its high adsorption efficiency and improved reusability. Overall, the isotherm results support two distinct adsorption behaviours: Sample A follows a Freundlich-type adsorption profile, reflecting heterogeneous binding on a chemically diverse carbonaceous surface, whereas Samples B, C, and D follow Langmuir-type behaviour, consistent with adsorption on more defined Fe-containing sites. These findings align with the kinetic results and indicate that iron incorporation changes the adsorption pathway from predominantly heterogeneous carbon–surface interactions toward stronger Fe-assisted surface binding. The Freundlich model provided the best description for Sample A (*K*_F_ = 10.26 ± 0.17; *n* = 4.31 ± 0.14; adjusted *R*^2^ = 0.996; SSE = 0.264), consistent with adsorption on a heterogeneous carbon surface. Samples B–D were better described by the Langmuir model according to the combined non-linear fitting criteria in Tables 4 and S4. The coexistence of Freundlich-type behaviour for A and Langmuir-type behaviour for the iron-containing materials should be interpreted as a difference in the apparent distribution of adsorption energies and accessible sites; it does not, by itself, establish a unique molecular adsorption mechanism. Freundlich and Langmuir models describe heterogeneous and idealized monolayer adsorption behaviour, respectively, but both are phenomenological models.^43,44^

To strengthen model selection beyond linearised *R*^2^ values, non-linear regression of the Langmuir and Freundlich isotherms was evaluated using reduced chi-square (*χ*^2^) and residual sum of squares (SSE) (Table S4). For Sample A, the Freundlich model yielded the lowest *χ*^2^ (0.07) and SSE (0.26) and the highest *R*^2^ (0.997), indicating that tetracycline adsorption on the iron-free *Acacia*-derived carbon occurs on energetically heterogeneous surfaces with potential multilayer formation, as commonly reported for biochar and activated carbon adsorbents. In contrast, for Samples B–D, the Langmuir model consistently provided lower *χ*^2^ and SSE and higher *R*^2^ than Freundlich, indicating monolayer-dominant adsorption at relatively homogeneous Fe(iii) coordination sites. The previously considered Redlich–Peterson fits, which produced non-physical exponents for some samples, were therefore discarded in favour of these statistically robust and mechanistically consistent Langmuir/Freundlich models. It is worth noting that the linearised Langmuir and Freundlich plots (Fig. S6) yield uniformly high *R*^2^ values for all samples, illustrating that linear regression alone can overestimate goodness-of-fit for transformed isotherm equations; our model selection therefore relies primarily on non-linear fitting with *χ*^2^ and SSE rather than on linearised *R*^2^ values.

Non-linear regression of the original Langmuir and Freundlich equations (Fig. 9) and the associated error analysis (*χ*^2^, SSE; Table S4) thus confirms that Sample A is best described by the Freundlich model, while Samples B–D are best described by the Langmuir model. Consistent with recent reports comparing linear and non-linear isotherm regression, the linearised plots initially suggested comparable Langmuir and Freundlich fits for Sample A; however, non-linear error analysis (*χ*^2^, SSE) demonstrated that Freundlich provides the superior description of the equilibrium data, whereas Langmuir remains the best non-linear model for the iron-modified Samples B–D.

Although the nonlinear Langmuir model yielded fitted *q*_m_ values of approximately 20 mg g^−1^ for Samples B–D, these values were obtained from equilibrium data collected over an initial TC concentration range of 10–40 mg L^−1^ at an adsorbent dosage of 1.0 g L^−1^. Under the highest initial concentration, the theoretical mass-balance maximum is 40 mg g^−1^. The fitted Langmuir capacities are therefore physically accessible for the isotherm dataset and should be interpreted as model-derived equilibrium parameters rather than as capacities measured under the fixed 10 mg L^−1^ condition. In contrast, the separate kinetic and optimisation experiments were conducted at *C*_0_ = 10 mg L^−1^ and an adsorbent dosage of 1.0 g L^−1^, for which the maximum physically attainable adsorption capacity is 10 mg g^−1^. Accordingly, all experimental and fitted kinetic capacities reported for those conditions were rechecked against this mass-balance limit.

Normalisation of *q*_max_ to BET surface area gives surface-specific capacities of 709, 1,892, 1,669, and 2114 µg m^−2^ for A, B, C, and D, respectively. This analysis shows that BET surface area alone does not govern per-gram TC uptake capacity in these systems and strongly suggests that surface chemistry and the density of high-affinity sites dominate over bulk textural parameters. In Sample A, FTIR and XPS indicate a carbonaceous matrix rich in oxygenated functional groups (C=O, O–H, C–O) and partially graphitized aromatic domains, which provide distributed π–π stacking and hydrogen-bonding sites for tetracycline's naphthacene ring system and amide/hydroxyl moieties. These numerous, moderate-energy interactions are consistent with the Freundlich isotherm and pseudo-first-order kinetics observed for A. In contrast, Samples B–D contain surface Fe(iii) oxyhydroxide phases confirmed by Fe 2p XPS, and their adsorption is better described by Langmuir isotherms and pseudo-second-order kinetics, in line with literature reports of inner-sphere Fe(iii)-tetracycline complexation on metal-modified sorbents.^34,45^ The higher *q*_max_/*S*_BET_ ratios of B–D are therefore consistent with a higher density of strongly binding Fe(iii) sites per unit surface area, which compensates for their lower overall BET surface area and leads to similar *q*_max_ values per gram under the concentration range studied.

### 3.9 Post adsorption XRD analysis

**Fig. 10:**
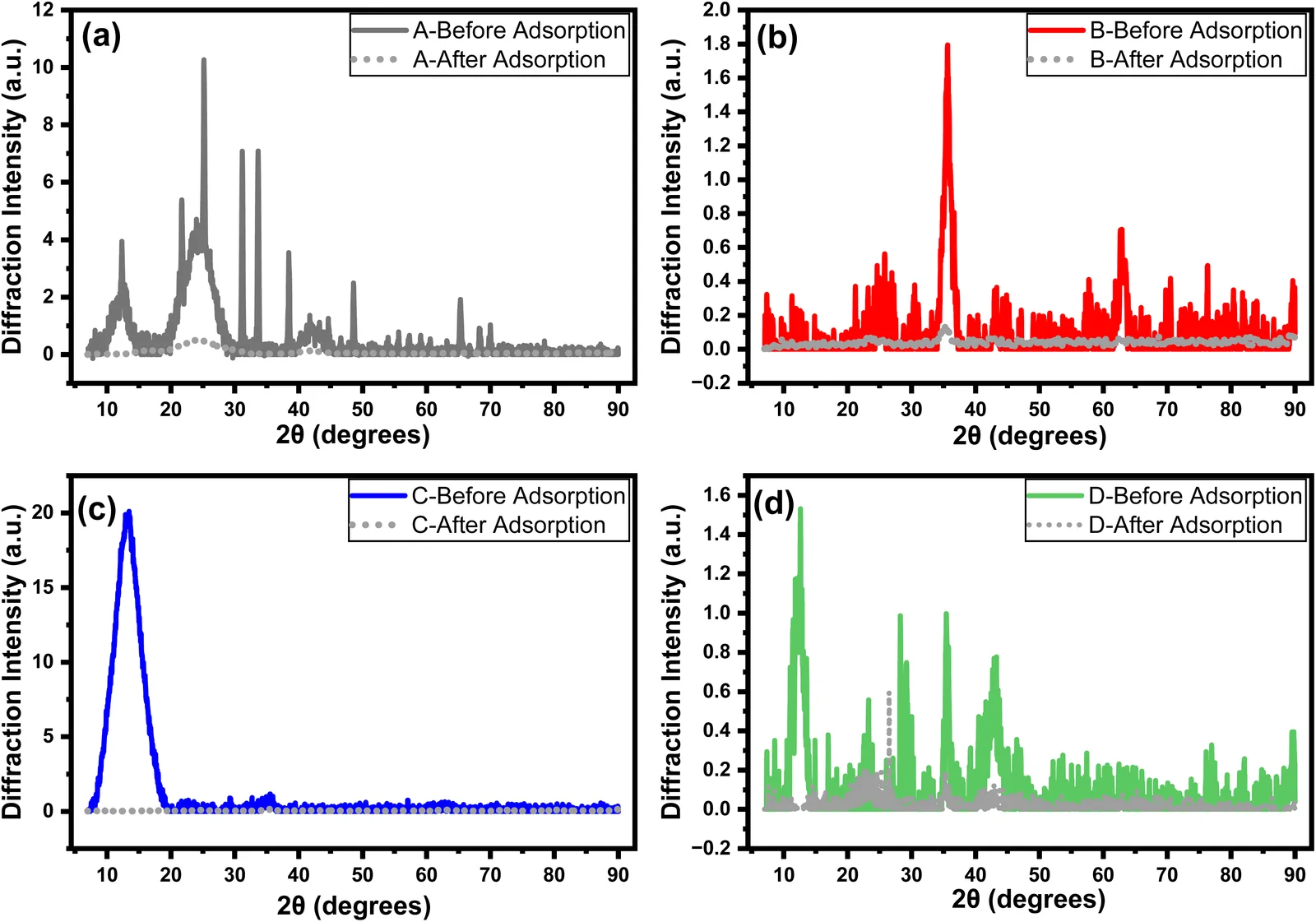
Powder X-ray diffraction (XRD) patterns of Samples A–D before and after tetracycline (TC) adsorption under the respective optimum adsorption conditions (*C*_0_ = 10 mg L^−1^, adsorbent dosage = 1.0 g L^−1^, 25 °C, optimum pH for each sample, and the experimentally determined equilibrium contact time). Panels (a–d) show Samples A–D, respectively. Solid lines represent the pristine adsorbents and dotted lines represent the corresponding materials after TC adsorption. Patterns were normalised for visual comparison; therefore, changes in absolute peak intensity should be interpreted qualitatively.

Powder X-ray diffraction (XRD) patterns were recorded for Samples A–D before and after tetracycline (TC) adsorption under the respective optimum adsorption conditions (*C*_0_ = 10 mg L^−1^, adsorbent dosage = 1.0 g L^−1^, 25 °C, and the optimum pH for each sample). The normalised diffraction patterns are presented in Fig. 10.

Before adsorption, Samples A–D exhibited distinct diffraction profiles, reflecting differences in precursor composition, thermal treatment and iron-modification route. Sample A showed a broad feature centred near 2*θ* ≈ 25°, which is consistent with the disordered carbon structure of the thermally treated *Acacia mearnsii*-derived material. The diffraction patterns of Samples B–D contained broad features and weak reflections associated with their compositionally different iron-modified carbonaceous structures. Because several features were broad and of low intensity, definitive phase assignment from XRD alone was not attempted.

After TC adsorption, the post-adsorption patterns retained the broad and/or weak features characteristic of the respective pristine materials. No additional sharp diffraction peaks attributable to a newly formed crystalline phase were observed within the detection limits of XRD.

The lower apparent intensity of several reflections after adsorption should be interpreted qualitatively. Because an internal standard was not used, apparent differences in diffraction intensity may be influenced by specimen packing, sample thickness, preferred orientation and the normalisation procedure. Accordingly, the post-adsorption XRD data do not permit a quantitative estimate of crystallinity loss or surface amorphisation.

Overall, the absence of additional sharp diffraction peaks indicates that no new crystalline reaction product was detected after TC adsorption. However, XRD cannot exclude amorphous, poorly crystalline or low-abundance surface deposits. The adsorption mechanism was therefore interpreted using the combined evidence from FTIR, XPS, pH-dependent adsorption behaviour and equilibrium modelling. The results are consistent with contributions from π–π interactions and hydrogen bonding for Sample A, and possible interactions between TC functional groups and Fe(iii)-containing surface sites for Samples B–D. Direct identification of a specific inner-sphere surface complex would require dedicated comparative post-adsorption spectroscopic measurements with appropriate controls.^41,42,46,47^

### 3.10 Preliminary regeneration performance

**Fig. 11:**
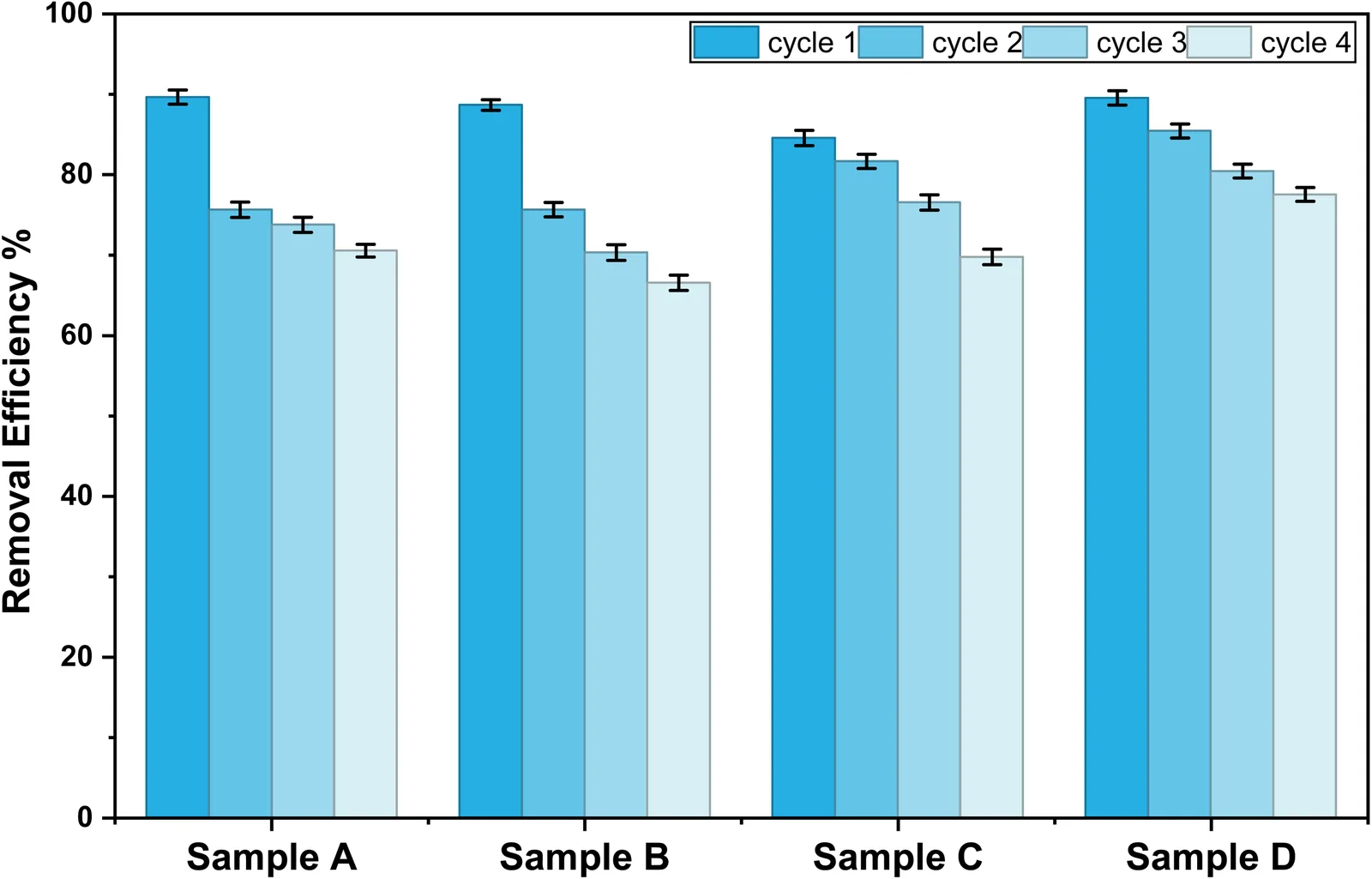
TC removal efficiency during four consecutive adsorption–desorption cycles for Samples A–D. Bars represent mean ± standard deviation of independent experiments. The values shown are absolute TC removal efficiencies for each cycle; they are not relative retention percentages. For Sample D, the fourth-cycle absolute removal efficiency was 77.5 ± 0.5%, corresponding to 86.5% relative retention of its first-cycle removal efficiency.

The regeneration performance of the four adsorbents was evaluated over four consecutive laboratory adsorption–desorption cycles (Fig. 11). After each adsorption cycle, the spent adsorbent was separated by filtration through a 0.45 µm membrane, washed three times with 10 mL of 0.1 M HCl, rinsed with deionized water until the rinse-water pH reached 6.5–7.0, and dried at 60 °C for 12 h before reuse under the same batch adsorption conditions.

All adsorbents showed a decrease in absolute TC removal efficiency during four consecutive adsorption–desorption cycles. Sample A decreased from 89.7% removal efficiency in the first cycle to 70.5% in the fourth cycle, corresponding to 78.6% relative retention of first-cycle removal performance and a 21.4% relative loss. Sample B decreased from 88.7% to 66.6%, corresponding to 75.1% relative retention and a 24.9% relative loss. Sample C decreased from 84.6% to 69.8%, corresponding to 82.5% relative retention and a 17.5% relative loss. Sample D decreased from 89.6 ± 0.9% in the first cycle to 77.5 ± 0.5% in the fourth cycle. Thus, 77.5% is the absolute removal efficiency measured in the fourth cycle, whereas the relative retention of the initial removal performance was 86.5%, corresponding to a 13.5% relative loss.^48^ The cumulative solid-mass recovery of Sample D after four cycles was 97.3 ± 0.8%, indicating that most of the adsorbent mass was physically recovered during the regeneration procedure. However, solid-mass recovery does not establish chemical stability or preservation of iron-containing surface phases. Adsorption capacity per cycle, TC desorption efficiency, dissolved Fe release, post-regeneration Fe speciation, surface functional groups, pore structure, and residual or released TC were not measured. Therefore, the present four-cycle experiment provides only preliminary laboratory-scale evidence of partial retention of TC-removal performance after repeated acid-washing cycles. The results should not be interpreted as evidence of long-term adsorbent stability, economic feasibility, scalability, or practical water-treatment applicability. Further work should include cycle-specific adsorption capacity, desorption efficiency, solid-mass recovery, Fe-leaching analysis, post-regeneration characterisation, and longer-term cycling under representative water matrices.^48–51^

### 3.11 Preliminary effect of added chloride, sulfate, and nitrate salts on TC removal

**Fig. 12:**
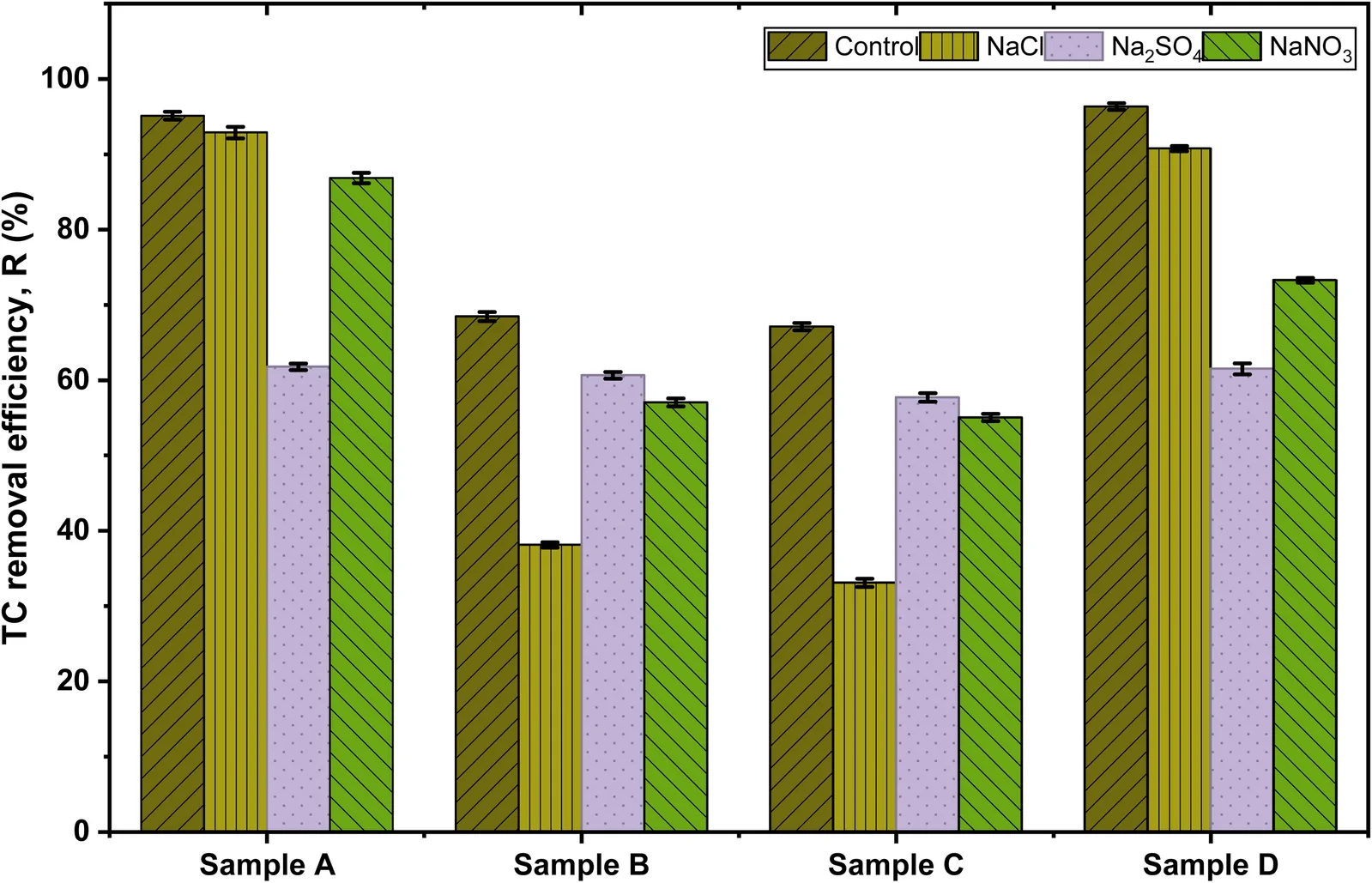
Preliminary effect of added salts on TC removal efficiency by Samples A–D. Control experiments contained no added salt. NaCl, Na_2_SO_4_, and NaNO_3_ were tested at a nominal concentration of 20 mg L^−1^. Final solution pH was not measured in the original added-salt experiments. Because the tested salts were not adjusted to equal molar concentration or matched ionic strength, the results are interpreted as preliminary nominal-concentration screening data.

The preliminary effect of added NaCl, Na_2_SO_4_, and NaNO_3_ on TC removal was evaluated relative to an anion-free control under the respective adsorption conditions for Samples A–D (Fig. 12). The salts were tested at a nominal concentration of 20 mg L^−1^. Because equal mass concentrations of NaCl, Na_2_SO_4_, and NaNO_3_ do not correspond to equal molar concentrations or matched ionic strengths, the experiment is interpreted as a nominal-concentration screening test rather than a controlled ionic-strength comparison. Initial solution pH was adjusted to the selected adsorption condition; however, final solution pH was not measured in the original added-salt experiments. Consequently, the contribution of salt-induced pH changes to the observed TC-removal differences cannot be separated from the effects of added salts.^52,53^ TC removal varied among the tested salt conditions and adsorbents. For Sample A, removal was 95.12 ± 0.52% for the anion-free control, 92.89 ± 0.76% with NaCl, 61.77 ± 0.43% with Na_2_SO_4_, and 86.85 ± 0.68% with NaNO_3_. For Sample B, the corresponding values were 68.45 ± 0.61%, 38.12 ± 0.36%, 60.64 ± 0.45%, and 57.06 ± 0.54%, respectively. For Sample C, removal was 67.12 ± 0.48%, 33.09 ± 0.54%, 57.71 ± 0.57%, and 55.05 ± 0.48%, respectively. For Sample D, removal was 96.35 ± 0.45% for the control, 90.76 ± 0.34% with NaCl, 61.51 ± 0.73% with Na_2_SO_4_, and 73.27 ± 0.32% with NaNO_3_. These results show that TC removal was sensitive to the tested added-salt conditions. However, because the tested salts were added at the same nominal mass concentration rather than at equal molar concentration or rigorously matched ionic strength, the observed differences cannot be assigned uniquely to anion identity, anion charge, counterion effects, ionic-strength effects, or pH-dependent changes in solution chemistry. The results should therefore be considered preliminary nominal-concentration screening data. Further experiments using specified salts at equal molar concentration or matched ionic strength, together with mixed-electrolyte and real-water matrices, are required before claims regarding anion selectivity or groundwater applicability can be made.

### 3.12 Comparative study

Comparison of Samples A–D provides insight into the influence of iron speciation, particle dispersion, and carbon support structure on adsorption performance, adsorption mechanisms, and regeneration behaviour. The adsorption performance of the developed materials was benchmarked against previously reported adsorbents for tetracycline removal (Table 5).

**Table 5:** Comparison of tetracycline adsorption performance of carbon-based and related adsorbents. The column ′*C*_0_ for *q*_max_ (mg L^−1^) denotes the initial concentration range used to determine the reported maximum adsorption capacity, which directly affects the magnitude of *q*_max_ obtained by isotherm modeling. The present study was conducted at environmentally relevant concentrations (10–40 mg L^−1^) reflecting reported TC levels in contaminated water bodies.^57^

Adsorbent	*q*_max_ (mg g^−1^)	Dosage (g L^−1^)	*C*_0_ for *q*_max_ (mg L^−1^)	Removal (%)	Contact time (h)	Mechanism	Reference
Molecularly imprinted polymer	416.5–666.5 (Langmuir, 293–323 K)	0.6	25–100 (individual TC concentration 25–100 mg L^−1^)	>90 at pH 5 under optimum conditions (60 min, 0.6 g L^−1^, 25 mg L^−1^)	1	Pseudo-second order	55
Grape marc activated carbon (700 °C)	17.38	1.5	50–200 (TC isotherm concentration range)	94	6	Pseudo-first order	54
Hydrous ferric oxide	70.59	1.0	10–70 (isotherm *C*_0_ range at 298 K)	88–95 (pH 3–7, 1.0 g L^−1^)	3.5–7	Pseudo-second order	42
Ca-modified corn straw biochar	93.46	1.0	10–100 mg L^−1^	85	24	Pseudo-second order	40
Zero-valent iron biochar	38.53	1.6	50–100	80–90	1.5	Pseudo-second order	56
Sample A	21.98	1.0	10–40	92.7	2	Pseudo-first order	This study
Sample B	22.51	1.0	10–40	88.7	6	Pseudo-second order	This study
Sample C	20.69	1.0	10–40	84.6	6	Pseudo-second order	This study
Sample D	21.77	1.0	10–40	90.0	6	Pseudo-first order	This study

Sample A, which followed pseudo-first-order kinetics and Freundlich isotherm behaviour, exhibited an optimized adsorption capacity of 21.98 mg g^−1^ under the selected operating conditions (pH 7.0, 1.0 g L^−1^ dosage, 2 h contact time). This performance is comparable to thermally modified grape marc activated carbons (17.38 mg g^−1^) and falls within the lower range reported for conventional activated carbon systems.^54^

Samples B, C, and D followed pseudo-second-order kinetics and were best described by the Langmuir isotherm model, yielding maximum adsorption capacities of 22.51, 20.69, and 21.77 mg g^−1^, respectively. Although these capacities are lower than those reported for highly engineered adsorbents such as molecularly imprinted polymers (416–667 mg g^−1^)^55^ and calcium-modified corn straw biochar (93.46 mg g^−1^),^40^ such materials are often evaluated under substantially different operating conditions, including elevated temperatures, prolonged equilibration times, or much higher initial tetracycline concentrations.

An important advantage of the present materials is their relatively rapid adsorption kinetics. Equilibrium was achieved within 2 h for Sample A and within 6 h for Samples B–D, which compares favourably with several reported adsorbents requiring considerably longer equilibration periods. For example, calcium-modified corn straw biochar requires approximately 24 h to reach equilibrium, while hydrous ferric oxide systems typically require around 7 h.^40^

Textural analysis further confirmed that adsorption performance cannot be explained by BET surface area alone. Normalisation of *q*_max_ to *S*_BET_ reveals surface-area-specific capacities of 709, 1,892, 1669 and 2114 µg m^−2^ for Samples A, B, C and D, respectively. The 2.4–3.0-fold higher surface-specific capacity of the iron-modified materials is consistent with a mechanistic shift from distributed physisorptive interactions in Sample A to more concentrated Fe(iii)-coordination chemistry in Samples B–D. This interpretation agrees with reports on iron-oxide-functionalised adsorbents, where surface metal site density, rather than BET surface area, is often the primary predictor of antibiotic uptake capacity. The direct comparison of three distinct iron-incorporation strategies (acidic precipitation, NH_4_OH-adjusted precipitation and *in situ* Fe(iii) oxide/oxyhydroxide immobilisation) on bio-derived carbon matrices within a single controlled experimental framework represents a key novelty of this study. Within this framework, *in situ* nanoparticle immobilisation (Sample D) yields a higher XPS satellite-to-main intensity ratio (*i*_ratio_ ≈ 0.18 *vs.* 0.13–0.14 for B and C), indicating a larger fraction of exposed Fe(iii) sites, and corresponds to the highest fitted Langmuir affinity constant among the iron-modified sorbents (*K*_L_ = 0.671 ± 0.116 L mg^−1^, *R*^2^ = 0.963) and the highest relative retention of first-cycle TC removal performance after four regeneration cycles (86.5%). These performance metrics, considered together rather than individually, define the main contribution of the present work.

As summarised in Table 5, molecularly imprinted polymers and Ca(OH)_2_-modified corn straw biochar reach *q*_max_ values of 416–667 and 93.46 mg g^−1^, respectively, but these capacities are obtained at much higher initial tetracycline concentrations (25–100 mg L^−1^ and up to 100 mg L^−1^) than those used in the present study. In contrast, Samples A–D were evaluated at *C*_0_ = 10–40 mg L^−1^, which is closer to reported environmental levels, and still show competitive capacities of ∼21 mg g^−1^ with favorable kinetics and regeneration performance.

Although several literature adsorbents exhibit higher reported maximum adsorption capacities, many of these values were obtained at substantially higher initial tetracycline concentrations, longer equilibrium times, elevated temperatures, or under conditions that are not directly comparable to those employed in this study. Under the present experimental conditions, Sample D provided the most favourable balance between adsorption efficiency, adsorption capacity, adsorption kinetics, and regeneration performance. It achieved 90.0% TC removal at a dosage of 1.0 g L^−1^ within 6 h while retaining 77.5% of its initial adsorption performance after four regeneration cycles. When normalized for dosage and contact time, its performance compares favourably with hydrous ferric oxide^42^ and zero-valent iron-loaded biochar systems.^56^

Overall, the results demonstrate that the cellulose-derived Fe nanoparticle composite (Sample D) offers the most balanced combination of adsorption performance, operational stability, and reusability, making it a promising candidate for practical tetracycline remediation applications.

## 4. Conclusions

Four compositionally distinct carbonaceous adsorbents were evaluated for tetracycline removal under controlled laboratory batch conditions. Sample A was derived from *Acacia mearnsii* extract, Samples B and C were nominally iron-modified *Acacia mearnsii*-derived carbons, and Sample D was a cellulose-derived carbon-fibre composite containing surface Fe(iii) oxide/oxyhydroxide species.

TC removal depended on solution pH, adsorbent dosage, contact time, precursor type, and measured surface characteristics. The non-linear pseudo-first-order model provided the best empirical description of the 20–120 min kinetic data for all samples, while intraparticle diffusion contributed to uptake but was not the sole rate-controlling process. The Freundlich model best described Sample A, whereas the Langmuir model provided the best fit for Samples B–D under the isotherm conditions. The fitted Langmuir capacities of approximately 20–21 mg g^−1^ are model-derived equilibrium parameters from the isotherm dataset collected at initial TC concentrations of 10–40 mg L^−1^ and do not represent capacities achieved under the separate fixed-concentration kinetic condition of *C*_0_ = 10 mg L^−1^.

XPS and SEM-EDS results support the presence of iron-associated surface species in Samples B–D, predominantly with Fe(iii) oxide/oxyhydroxide character at the surface. These species may contribute to TC adsorption; however, the present data do not establish a unique molecular adsorption mechanism or direct inner-sphere coordination. TC removal refers to the decrease in TC concentration in the filtered aqueous phase and does not demonstrate TC degradation or mineralisation.

The four-cycle regeneration experiment showed partial retention of TC-removal performance. Sample D achieved an absolute removal efficiency of 77.5 ± 0.5% in the fourth cycle, corresponding to 86.5% relative retention of its first-cycle removal efficiency. The observed 97.3 ± 0.8% solid-mass recovery indicates physical retention of the adsorbent mass but does not establish chemical stability. Dissolved Fe release, post-regeneration Fe speciation, surface chemistry, pore structure, and TC desorption or release were not measured. This result provides preliminary laboratory-scale evidence of reusability but does not establish long-term chemical stability, cost-effectiveness, scalability, or applicability in real-water treatment.

This work provides a laboratory-scale comparison of compositionally distinct biomass-derived carbonaceous adsorbents in synthetic TC solutions. Because the materials differ in precursor composition, carbonisation history, morphology, porosity, and nominal iron formulation, the observed performance differences cannot be attributed solely to iron incorporation. Future studies should include iron-free cellulose-derived controls, matched carbonisation conditions, bulk Fe quantification, iron-leaching analysis, post-regeneration characterisation, real-water matrices, and continuous-flow experiments before individual synthesis variables or practical water-treatment potential can be assessed.

## Author contributions

Elham Jalali: writing – original draft, visualization, validation, software, resources, project administration, methodology, investigation, funding acquisition, formal analysis, data curation, conceptualization. Elizabeth Erasmus: writing – review & editing, visualization, validation, supervision, software, resources, project administration, methodology, investigation, formal analysis, data curation, conceptualization. Shahab Maghsoudi: writing – review & editing, methodology, investigation. Marietjie Schutte-Smith: writing – review & editing, validation, investigation. Ivan Novak: writing – review & editing, Synthesis and preparation of the adsorbent material, and Hendrik. G. Visser: writing – review & editing, visualization, validation, supervision, resources, project administration, methodology, investigation, formal analysis, data curation, conceptualization.

## Conflicts of interest

The authors declare that they have no known competing financial interests or personal relationships that could have appeared to influence the work reported in this paper.

## Supplementary Material

RA-OLF-D6RA06684D-s001

## Data Availability

The data supporting this article are available in the supplementary information (SI). Supplementary information: the particle size distribution of Sample A, SEM-EDS spectra, high-resolution C 1s, O 1s, and N 1s XPS spectra with peak deconvolution and fitted parameters, linearized and residual isotherm plots, complete non-linear isotherm and kinetic (pseudo-first-order, pseudo-second-order, and intraparticle-diffusion) fitting parameters with goodness-of-fit statistics, pH-matched UV-vis calibration curves and analytical validation data, and the initial and final solution pH values recorded after equilibration (Fig. S1–S8 and Tables S1–S8). See DOI: https://doi.org/10.1039/d6ra06684d.
