# Supplementary material for: Comparative tetracycline removal by Acacia mearnsii-derived carbons and a cellulose-derived iron oxide/oxyhydroxide carbon composite
Source: RSC Adv. 2026 Sep 1;16(42):51515–36. doi: 10.1039/d6ra06684d (PMC13531595; doi:10.1039/d6ra06684d)
Supplement: Supplementary file 1 [file RA-016-D6RA06684D-s001.pdf]

Supplementary Information (SI) for RSC Advances.
This journal is © The Royal Society of Chemistry 2026

Supplementary information

Comparative Tetracycline Removal by *Acacia mearnsii*-Derived Carbons and a Cellulose-Derived Iron Oxide/Oxyhydroxide Carbon Composite

Elham Jalali[1,2], Elizabeth Erasmus[1], Shahab Maghsoudi[2], Marietjie Schutte-Smith[1], Ivan Novak[3], and Hendrik. G. Visser[1]*

[1]Chemistry Department, University of the Free State, Bloemfontein, 9300 South Africa

[2] Department of Chemistry, Faculty of Science, Shahid Bahonar University of Kerman, Kerman, Iran

[3] Ivan Novak, Pod Rovnicami 7, 84104 Bratislava, Slovakia

Corresponding author: * visserhg@ufs.ac.za (H.G. Visser email)

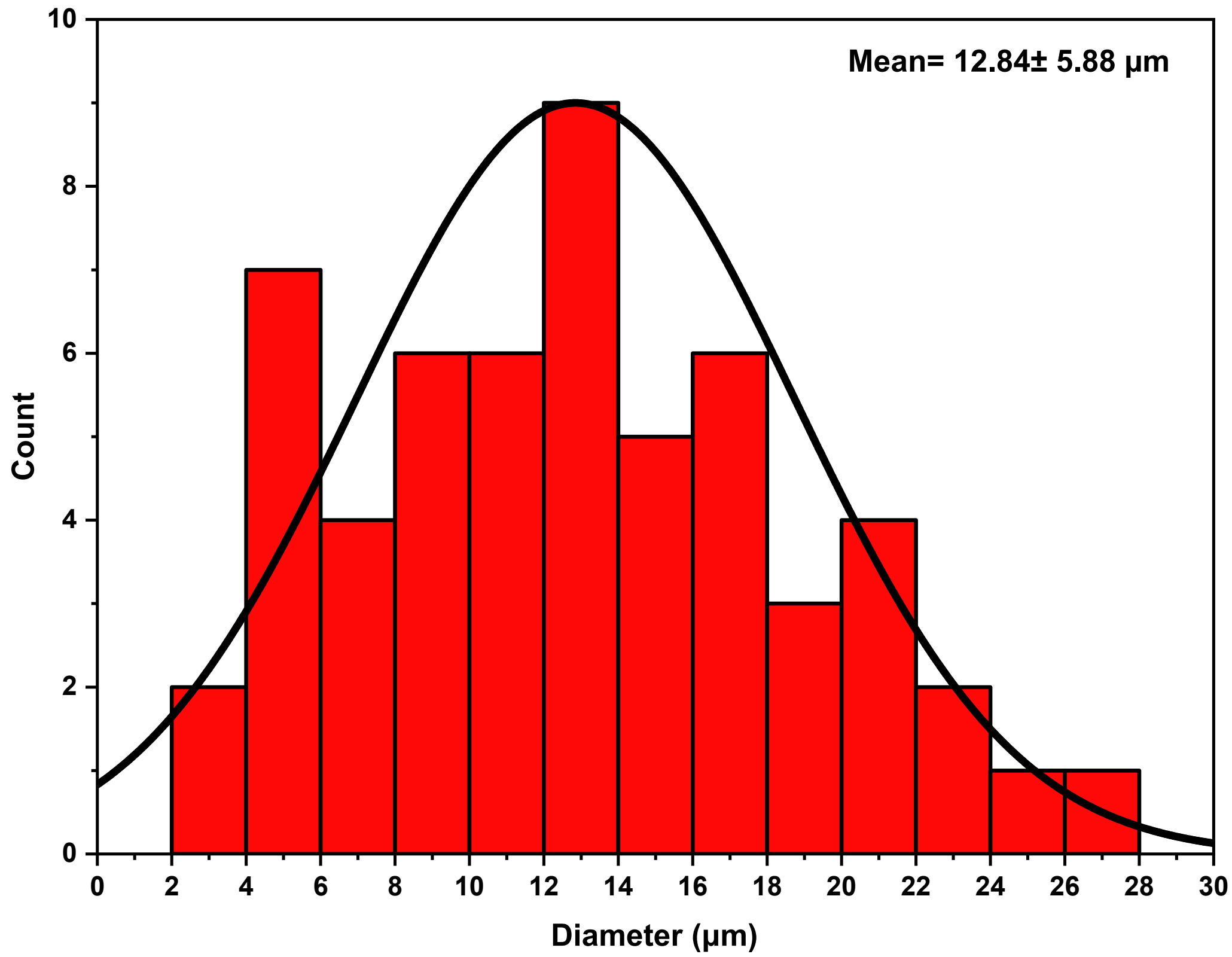

Figure. S1. Particle size distribution of Sample A (thermally modified *Acacia mearnsii* extract), showing a histogram of particle diameters with a mean size of 12.8 ± 5.8 µm

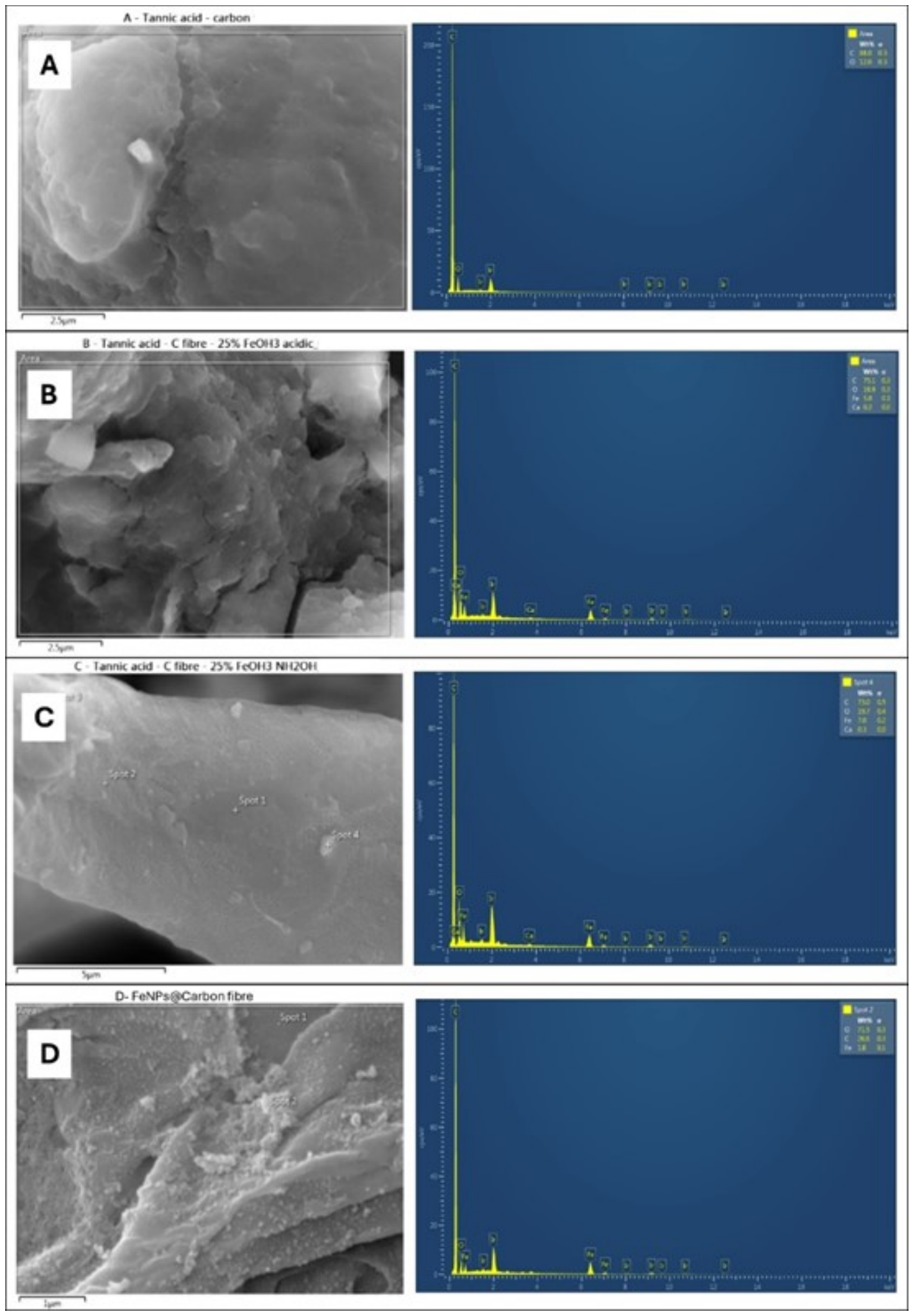

Fig. S2. EDS spectrum of the four different samples: (A) Acacia mearnsii (Black wattle) extract thermally modified, (B) Acacia mearnsii (Black wattle) extract, thermally modified with 25% $Fe(OH)_3$ added under acidic conditions (C) Acacia mearnsii (Black wattle) extract, thermally modified, with 25% $Fe(OH)_3$ and pH adjusted using NH_4OH and (D) Fe(III) oxide/oxyhydroxide phases immobilized on delignified cellulose which was also heat treated to provide a highly structured fibrous morphology.

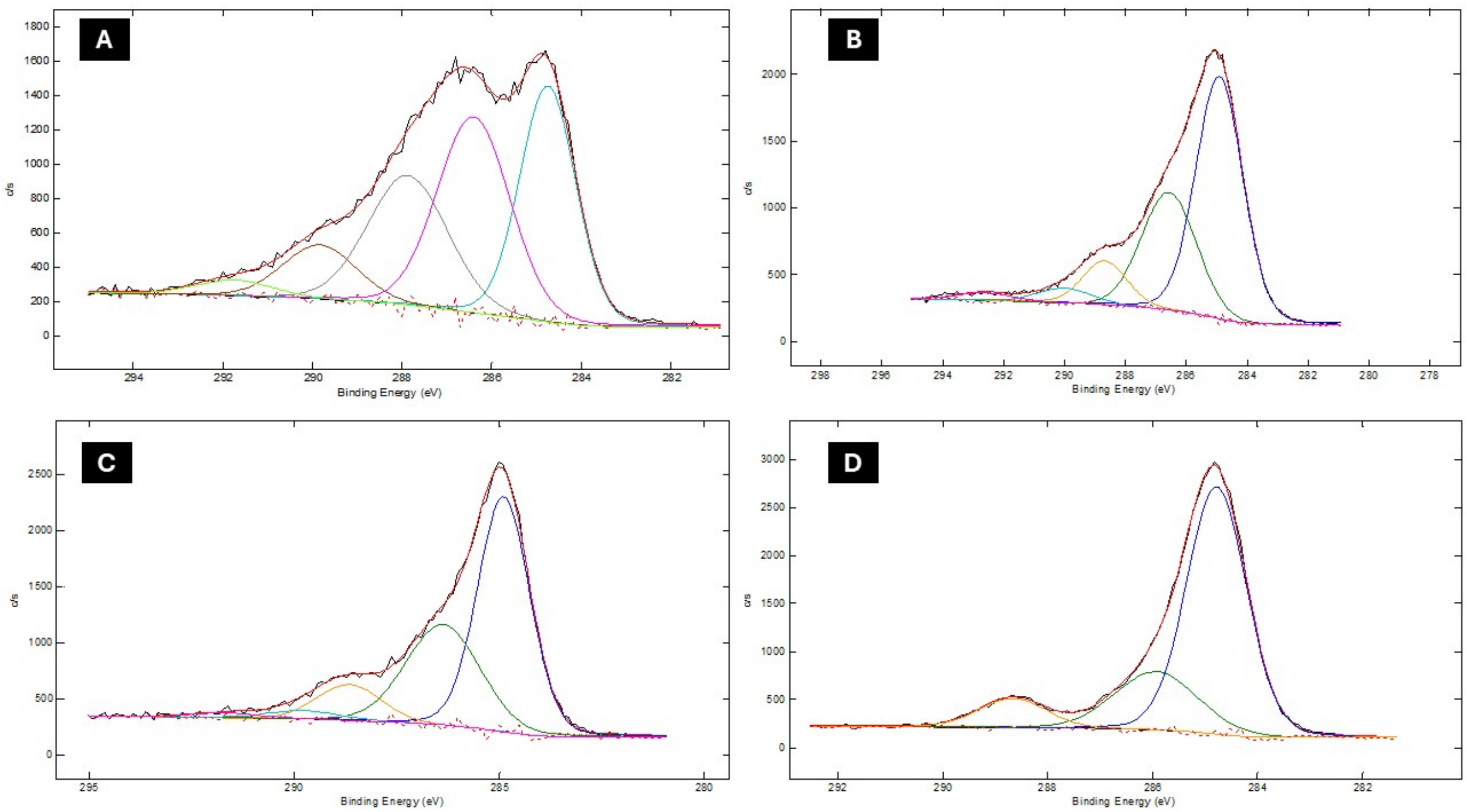

Fig. S3. High-resolution C 1s spectra with peak deconvolution. (A) Sample A shows five components: sp² C–C (284.75 eV), C–O (286.40 eV), C=O (287.87 eV), O–C=O (289.85 eV), and π–π* satellite (291.77 eV). (B, C) Iron-modified samples exhibit increased sp² content (~55%) consistent with iron-catalysed graphitisation. (D) Cellulose-based Sample D dominated by C–O bonding (~91%). Experimental data (black), envelope fit (red), individual peaks (colors), baseline (dashed)

C 1s High-Resolution Spectra (Supplementary Fig. S3)

High-resolution C 1s spectra (Supplementary Fig. S1) for all four samples were deconvoluted using Shirley background subtraction and mixed Gaussian–Lorentzian peak profiles. The peak assignments and relative area percentages are summarised in Table S1.

Table S1. C 1s XPS peak positions and relative area percentages for Samples A-D.

Peak	Assignment	Sample A (eV / %)	Sample B (eV / %)	Sample C (eV / %)	Sample D (eV / %)
C1	C–C / C=C (sp² aromatic, graphitic)	284.75 / 30.8	284.92 / 55.0	~285.0 / dominant	284.78 / 68.7

C2	C–O (hydroxyl, ether, phenolic)	286.40 / 33.2	286.57 / 29.8	~286.5	285.94 / 22.2
C3	C=O (carbonyl, quinone)	287.87 / 24.0	288.67 / 9.3	~288.7	288.67 / 9.1
C4	O–C=O (carboxyl, ester)	289.85 / 9.3	289.99 / 3.8	~290.0	—
C5	π–π* shake-up satellite	291.77 / 2.8	292.63 / 2.1	~292.5	—

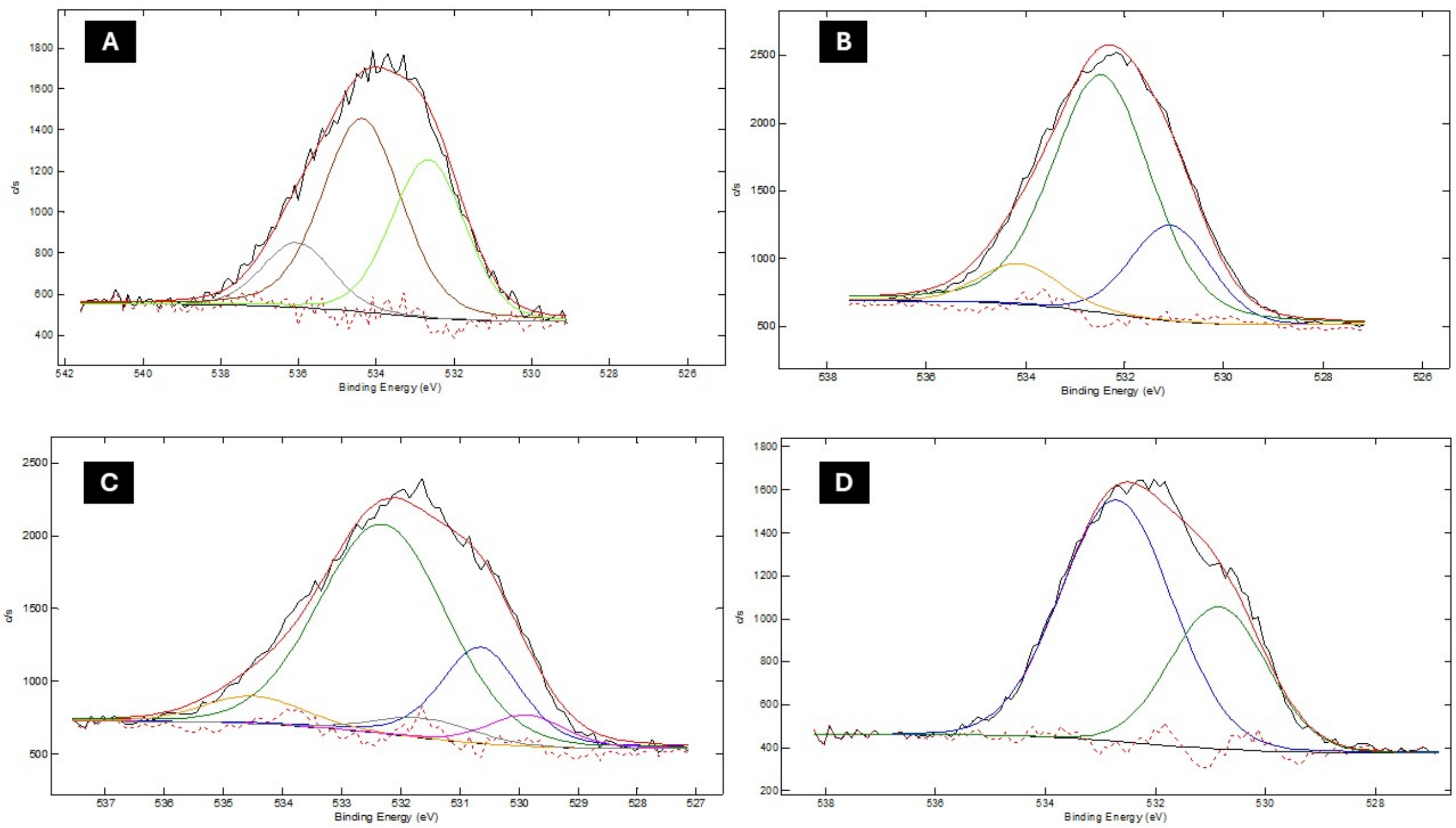

Fig. S4. High-resolution O 1s spectra with deconvolution. (A) Sample A shows C–OH (~531.5 eV) and C=O/C–O–C (~533 eV) only. (B–D) Iron-modified samples display three peaks: O1 (~530 eV, Fe–O lattice oxygen—arrow), O2 (~531.5 eV, Fe–OH/C–OH surface hydroxyl), and O3 (~533 eV, organic C=O/C–O–C). The Fe–O peak at 530 eV, absent in Sample A, confirms iron oxide formation. Experimental data (black), envelope fit (red), components (colors), baseline (dashed).

Sample A exhibited the richest oxygen functional group distribution, with C–O (33.2%) and C=O (24.0%) as the dominant components alongside a minor O–C=O carboxyl fraction (9.3%), characteristic of a biochar produced under moderate pyrolysis conditions with retained oxygenated surface chemistry. A π–π* satellite at 291.77 eV (2.8% area) confirms the presence of aromatic sp^2 carbon domains and conjugated π-electron systems in the graphitic microstructure of the wattle biochar matrix. Iron-modified samples B and C display a substantial increase in relative sp^2 C–C/C=C content (C1: ~55% for B vs. 31% for A), consistent with iron-catalysed graphitisation during thermal treatment at 400°C and concurrent occupation of oxygen-bearing surface sites by iron oxide species. Sample D is dominated by aromatic sp^2 carbon (C1 = 68.7%), consistent with the graphitic character developed during high-temperature (700 °C) carbonisation of the cellulose-derived carbon-fibre scaffold, with a smaller hydroxyl/ether contribution (C2 = 22.2%) retained from residual oxygenated surface groups.

O 1s High-Resolution Spectra (Supplementary Fig. S4)

O 1s spectra (Supplementary Fig. S2) were deconvoluted into two components for Sample A and three components for Samples B, C, and D, consistent with the established literature model for

iron-modified carbonaceous materials. The three-component model was preferred over higher-order fits because each peak can be unambiguously assigned to a chemically distinct oxygen environment, thereby avoiding overfitting artefacts that would introduce physically meaningless spectral components. Peak O1 at approximately 530 eV, assigned to lattice oxygen in Fe–O bonds (Fe_2O_3 or FeOOH), is entirely absent in Sample A but clearly resolved in Samples B, C, and D (Table S2). This low-binding-energy component, characteristic of metal oxide lattice oxygen (529–530 eV, as tabulated in standard XPS reference databases),constitutes direct spectroscopic evidence of iron oxide phase formation and distinguishes the Fe–O environment from all C–O species that appear exclusively above 531.5 eV. Peak O2 at ~531.5 eV encompasses surface hydroxyl groups (Fe–OH and C–OH), which represent the primary Brønsted base sites for proton exchange and hydrogen-bond donation during TC adsorption. It is explicitly noted that this peak should not be misassigned to oxygen vacancies, as ex situ XPS cannot detect oxygen vacancies in air-equilibrated powders; the signal at 531–532 eV in ambient-condition measurements invariably reflects adsorbed hydroxyls. Peak O3 at ~533 eV is attributed to organic oxygen functionalities (C=O carbonyl and C–O–C ether/glycosidic linkages) derived from the biochar or cellulose matrix. The relative area of O1 (Fe–O) is visually larger in Sample C than in Sample B (Supplementary Fig. S2), suggesting that the NH_4OH-mediated synthesis route produces a higher surface density of Fe–O bonds. This may reflect a more uniform surface precipitation of $Fe(OH)_3$ at the near-neutral pH imposed by NH_4OH, in contrast to the localised acidic deposition conditions employed in Sample B synthesis. Quantitative deconvolution of the O 1s region for all four samples is reported in Table S2.

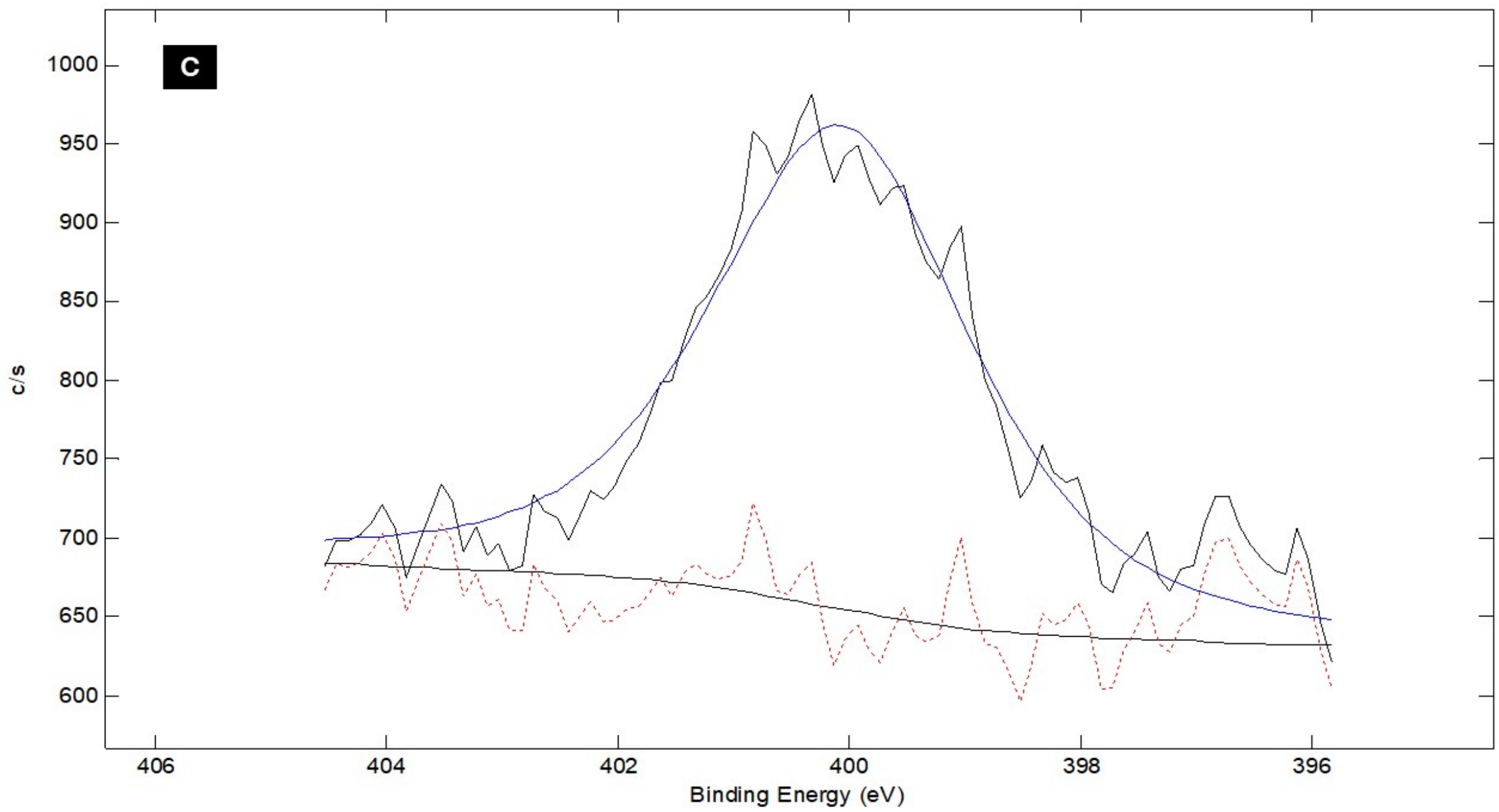

Fig. S5. High-resolution N 1s spectrum of Sample C showing a single peak at ~400 eV, assigned to surface NH_4^+ from NH_4OH-mediated synthesis. This nitrogen functionality provides additional hydrogen-bonding sites for tetracycline adsorption. Experimental data (black), fit (red), baseline (dashed)

N 1s Spectrum - Sample C (Supplementary Fig. S5)

The N 1s spectrum of Sample C (Supplementary Fig. S3) reveals a single broad peak centred at approximately 400 eV, fitted with one component and a Shirley background. A binding energy of ~400 eV is consistent with ammonium (NH_4^+) or amine (–NH_2/–NH–) nitrogen environments on the surface. Given the NH_4OH precipitation route employed for Sample C, the most parsimonious assignment is residual surface-bound NH_4^+ that was not removed during washing. The peak signal-to-noise ratio is modest, and the absence of a tabulated peak area value from the fitting output is a noted limitation. Nevertheless, the presence of nitrogen functionality is significant: protonated amine groups (–NH_3^+) at the adsorption-relevant pH of 5.0 can engage in electrostatic interactions with the anionic carboxylate and enolate groups of TC, providing an additional adsorption driving force not present in Sample B. This mechanistic distinction is consistent with the marginally lower optimal pH (5.0 vs. 5.0 for both B and C) but enhanced removal efficiency of Sample C relative to Sample B at equivalent adsorbent dosages.

Table S2. O 1s XPS peak deconvolution for adsorbents A–D. Binding energies (BE) and relative areas of each O 1s component were obtained from peak fitting; area percentages are expressed relative to the total O 1s intensity for each sample.

Component	Assignment	BE (eV)	Sample A (%)	Sample B (%)	Sample C (%)	Sample D (%)
**O1**	Fe–O (iron oxide lattice)	~530.0	—	22.3 (531.0)	22.4 (530.4)	16.4 (530.4)
**O2**	Fe–OH (surface oxyhydroxide)	~531.3	—	26.2 (531.8)	25.7 (531.6)	—
**O3**	C=O / organic oxygen	~532.5	44.5	21.1 (532.5)	36.4 (532.5)	47.1 (531.8)
**O4**	C–O–C / adsorbed water	~533.5	55.5	30.4 (533.4)	15.6 (533.9)	36.5 (533.2)

The O 1s fit for Sample D did not resolve a separate Fe–OH (O2) component; residual surface hydroxyl character for this sample is instead captured within the O3 (organic oxygen) contribution. Statements regarding Fe(III) oxyhydroxide site density for Sample D are therefore supported primarily by the Fe 2p satellite-to-main intensity ratio (Table 2, main text) rather than by O 1s deconvolution. Minor shifts in O1 binding energy (530.4–531.0 eV) across Samples B–D likely reflect small differences in local Fe coordination environment among the acidic (B), NH4OH-adjusted (C), and in situ nanoparticle (D) synthesis routes.

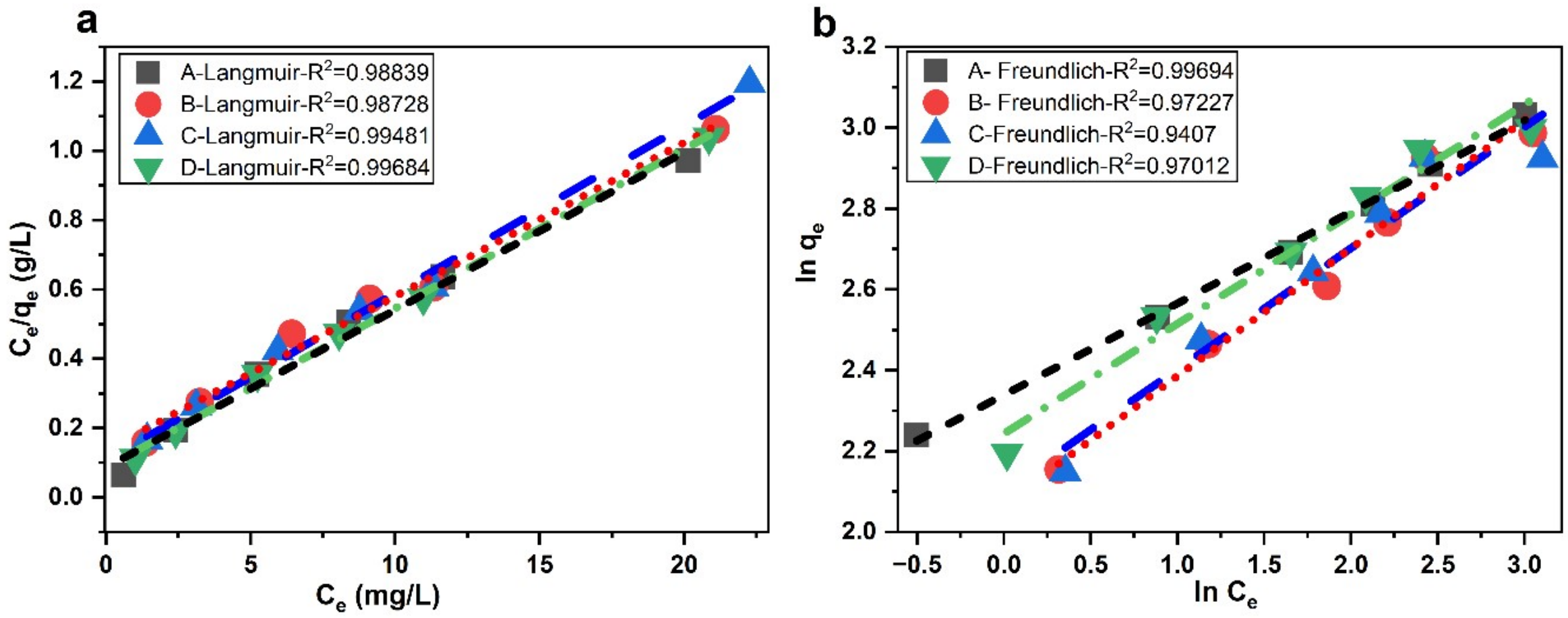

Figure S6. Linearised equilibrium isotherm plots for tetracycline (TC) adsorption onto Samples A–D. (a) Langmuir plots of C_e/q_e versus C_e and (b) Freundlich plots of $\ln q_e$ versus $\ln C_e$. Symbols represent experimental data and lines represent linear regression fits; the corresponding R^2 values are shown in the legends. These plots are provided for comparison with the non-linear isotherm fits presented in the main text and are not used for final model selection.

Table S3. Complete non-linear Langmuir and Freundlich isotherm fitting parameters for TC adsorption onto Samples A–D.

**Model**	**Sample**	q_m **(mg g⁻¹) or** K_F	K_L **(L mg⁻¹) or** n	R^2	**Adjusted** R^2	**Reduced** χ^2	**SSE**
**Langmuir**	A	$q_m = 19.5 \pm 1.4$ mg g⁻¹	$K_L = 1.03 \pm 0.41$ L mg⁻¹	0.834	0.793	3.45	13.8
**Langmuir**	B	$q_m = 20.3 \pm 1.4$ mg g⁻¹	$K_L = 0.449 \pm 0.120$ L mg⁻¹	0.920	0.900	1.55	6.20
**Langmuir**	C	$q_m = 20.8 \pm 1.0$ mg g⁻¹	$K_L = 0.439 \pm 0.082$ L mg⁻¹	0.959	0.949	0.817	3.27
**Langmuir**	D	$q_m = 20.5 \pm 0.8$ mg g⁻¹	$K_L = 0.671 \pm 0.116$ L mg⁻¹	0.963	0.954	0.745	2.98
**Freundlich**	A	$K_F = 10.3 \pm 0.2$	$n = 4.31 \pm 0.14$	0.997	0.996	0.066	0.264
**Freundlich**	B	$K_F = 8.61 \pm 0.99$	$n = 3.75 \pm 0.69$	0.900	0.875	1.94	7.75
**Freundlich**	C	$K_F = 8.78 \pm 0.95$	$n = 3.74 \pm 0.65$	0.909	0.886	1.81	7.24

**Freundlich**	D	$K_F = 10.0 \pm 0.8$	$n = 4.25 \pm 0.62$	0.934	0.918	1.32	5.29

Note: q_m and K_L are Langmuir parameters, whereas K_F and n are Freundlich parameters. All models were fitted by non-linear regression of untransformed q_e-versus-C_e data. The Langmuir parameters for Sample A are included for comparison only because the Freundlich model provides the preferred description of the Sample A equilibrium data.

Table S4. Complete non-linear kinetic and intraparticle-diffusion fitting parameters for TC adsorption onto Samples A–D.

**Sample**	**PFO** q_e **(mg g⁻¹)**	**PFO** k_1 **(min⁻¹)**	**PFO** R^2	**PFO SSE**	**PSO** q_e **(mg g⁻¹)**	**PSO** k_2 **(g mg⁻¹ min⁻¹)**	**PSO** h **(mg g⁻¹ min⁻¹)**	**PSO** R^2	**PSO fit status**	k_{id} **(mg g⁻¹ min⁻¹/²)**	C **(mg g⁻¹)**	**IPD** R^2
**A**	9.22 ± 0.37	0.0139 ± 0.0010	0.992	0.249	10.00[a]	0.00189 ± 0.00011	0.189	0.939	Constrained; unsuccessful	0.843 ± 0.042	−1.480 ± 0.348	0.979
**B**	6.57 ± 0.16	0.0350 ± 0.0031	0.950	0.638	8.15 ± 0.48	0.00456 ± 0.00116	0.303	0.909	Successful	0.473 ± 0.084	1.730 ± 0.701	0.780
**C**	6.61 ± 0.12	0.0277 ± 0.0015	0.986	0.207	8.59 ± 0.34	0.00304 ± 0.00046	0.225	0.975	Successful	0.544 ± 0.055	0.826 ± 0.461	0.915
**D**	9.48 ± 0.31	0.0139 ± 0.0008	0.995	0.174	10.00[a]	0.00202 ± 0.00012	0.202	0.934	Constrained; unsuccessful	0.850 ± 0.034	−1.372 ± 0.286	0.986

PFO and PSO equations were fitted directly to the untransformed q_t-versus-time dataset. Under the kinetic conditions ($C_0 = 10$ mg L⁻¹, $V = 0.010$ L, $m = 0.010$ g), the physical batch mass-balance maximum was 10 mg g⁻¹. The PSO fits for Samples A and D were constrained at this limit because unconstrained regression produced physically impossible equilibrium capacities. These constrained fits were not selected as preferred models.

Figure S7. Residual plots for non-linear Langmuir and Freundlich isotherm fitting of tetracycline adsorption data: (a, b) Sample A, (c, d) Sample B, (e, f) Sample C, and (g, h) Sample D. Residuals were calculated as $q_{e,exp} - q_{e,calc}$. The dashed horizontal line represents zero residual.

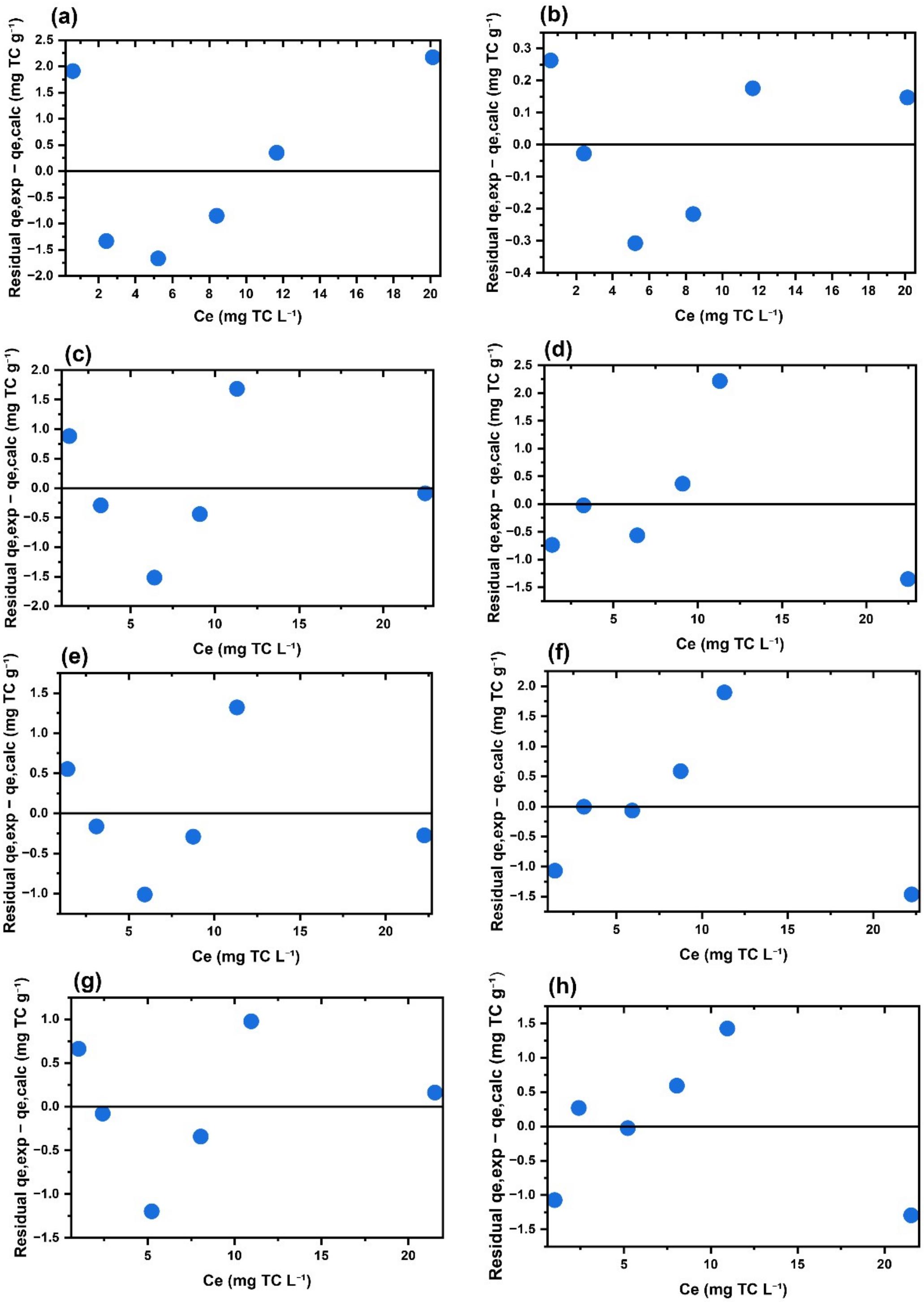

Table S5. Complete non-linear pseudo-first-order (PFO) and pseudo-second-order (PSO) kinetic fitting parameters for tetracycline adsorption onto Samples A–D.

Model	Sample	q_e(mg	Rate constant	h(mg g^{-1}	R^2	Adjusted	SSE	Reduced

		g⁻¹)		min⁻¹)		R^2		χ^2
**PFO**	A	9.22 ± 0.37	$k_1 = 0.0139 \pm 0.0010$ min⁻¹	—	0.992	0.991	0.249	0.0276
**PFO**	B	6.57 ± 0.16	$k_1 = 0.0350 \pm 0.0031$ min⁻¹	—	0.950	0.945	0.638	0.0709
**PFO**	C	6.61 ± 0.12	$k_1 = 0.0277 \pm 0.0015$ min⁻¹	—	0.986	0.984	0.207	0.0230
**PFO**	D	9.48 ± 0.31	$k_1 = 0.0139 \pm 0.0008$ min⁻¹	—	0.995	0.994	0.174	0.0193
**PSO**	A	10.00*	$k_2 = 0.00189 \pm 0.00011$ g mg⁻¹ min⁻¹	0.189	0.939	0.939	1.958	0.196
**PSO**	B	8.15 ± 0.48	$k_2 = 0.00456 \pm 0.00116$ g mg⁻¹ min⁻¹	0.303	0.909	0.899	1.168	0.130
**PSO**	C	8.59 ± 0.34	$k_2 = 0.00304 \pm 0.00046$ g mg⁻¹ min⁻¹	0.225	0.975	0.973	0.354	0.0394
**PSO**	D	10.00*	$k_2 = 0.00202 \pm 0.00012$ g mg⁻¹ min⁻¹	0.202	0.934	0.934	2.154	0.215

Note: The PFO and PSO models were fitted directly to untransformed q_t-versus-time data using non-linear regression. Values after ± indicate standard errors of the fitted parameters. $k_1$and $k_2$are the PFO and PSO rate constants, respectively; $h$is the initial adsorption rate, calculated as $h = k_2 q_e^2$. SSE denotes the residual sum of squares. Under the kinetic conditions ($C_0 = 10$ mg L⁻¹, $V = 0.010$L, $m = 0.010$g), the physical mass-balance adsorption limit was 10 mg g⁻¹. *For Samples A and D, the PSO $q_e$value was constrained to 10 mg g⁻¹ because unconstrained fitting returned non-physical equilibrium capacities. The PFO model was selected as the preferred kinetic model for all samples.

Table S6. pH-matched UV–Vis calibration parameters for tetracycline quantification during pH-dependent adsorption experiments.

Initial pH	TC concentration range (mg L⁻¹)	Calibration equation	R^2	Adjusted R^2
**3**	0.015–10.0	$A = 0.03873C + 0.00121$	0.99861	0.99854
**5**	0.015–10.0	$A = 0.03819C + 0.00117$	0.99861	0.99854
**7**	0.015–10.0	$A = 0.03710C + 0.00110$	0.99862	0.99854
**9**	0.015–10.0	$A = 0.03530C + 0.00103$	0.99862	0.99854
**11**	0.015–10.0	$A = 0.03238C + 0.00093$	0.99862	0.99854

In the calibration equations, A is the measured absorbance and C is the tetracycline concentration in mg L^{-1}. Calibration standards were prepared at the same initial pH as the corresponding pH-dependent adsorption experiments. UV–Vis measurements were performed at 357 nm. Raw calibration data are supplied in the accompanying ESI spreadsheet.

Table S7. Validation parameters for pH-specific UV–Vis determination of tetracycline at 357 nm.

Nominal pH	Membrane-filter recovery (%)	Matrix spike recovery (%)	LOD (mg L-1)	LOQ (mg L^{-1})	Adsorbent-blank absorbance at 357 nm
**3.0**	98.5 ± 0.8	97.4 ± 1.2	0.021	0.064	0.003
**5.0**	99.1 ± 0.6	98.8 ± 1.0	0.023	0.070	0.002
**7.0**	99.6 ± 0.4	101.2 ± 0.9	0.026	0.079	0.003
**9.0**	98.8 ± 0.7	99.5 ± 1.4	0.031	0.094	0.004
**11.0**	98.4 ± 0.9	96.8 ± 1.5	0.038	0.115	0.005

Note: Membrane-filter and matrix spike-recovery values are reported as mean ± standard deviation. Confirm the number of replicate determinations and add "$n = 3$" if each value is based on three independent measurements. LOD and LOQ were calculated using $LOD = 3.3\sigma/S$and $LOQ = 10\sigma/S$, where $\sigma$is the standard deviation of the analytical response and $S$is the slope of the pH-specific calibration curve.

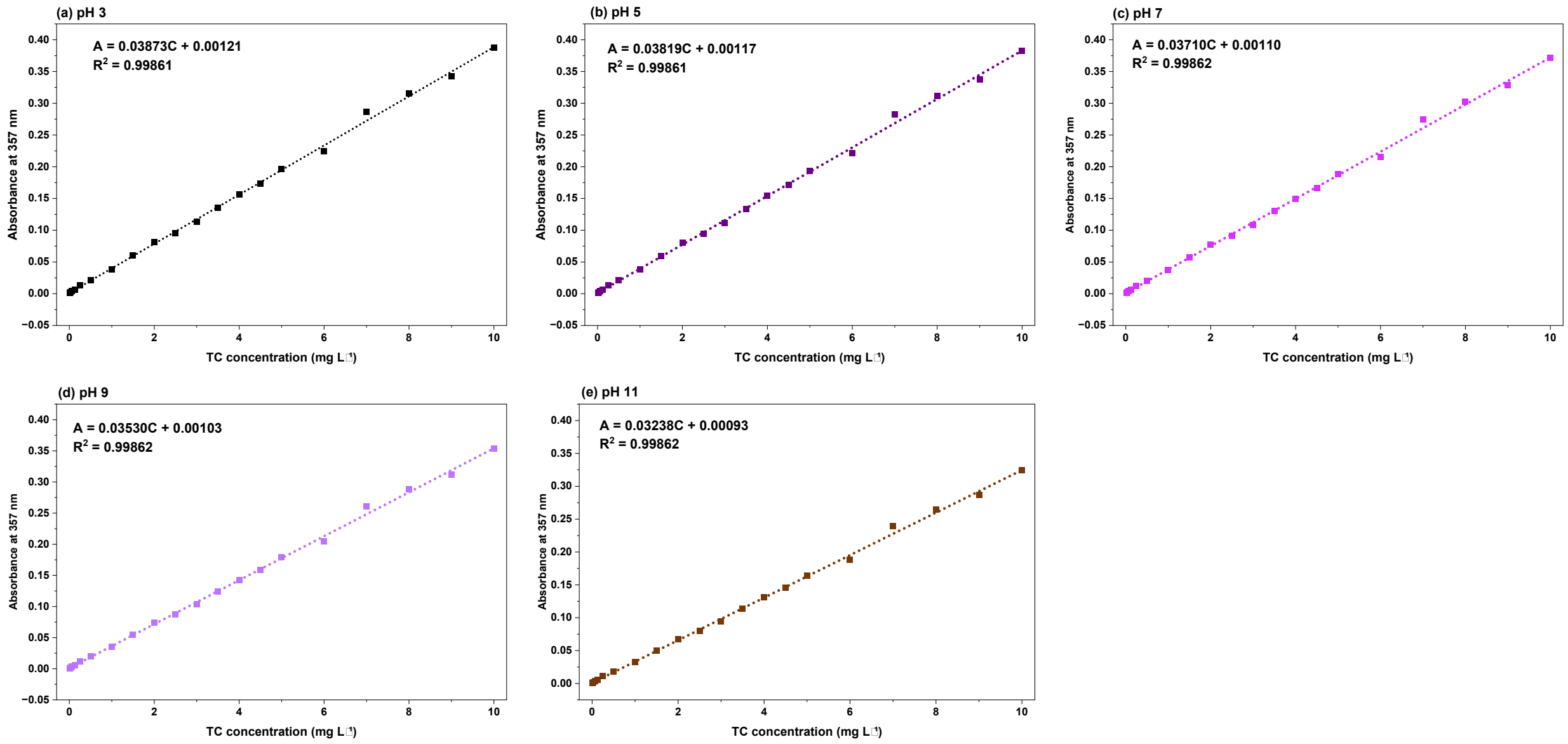

Figure S8. pH-matched UV–Vis calibration curves for tetracycline at (a) pH 3, (b) pH 5, (c) pH 7, (d) pH 9, and (e) pH 11. Calibration standards covered 0.015–10.0 mg L^{-1} and were prepared using the same pH-adjustment procedure as the corresponding adsorption experiments. Symbols represent measured absorbances, and solid lines represent linear regression fits. The regression equations and coefficients of determination are given in Table S6.

Table S8. Initial and final pH values of TC adsorption solutions after equilibration with Samples A–D.

Initial pH	Final pH: Sample A	Final pH: Sample B	Final pH: Sample C	Final pH: Sample D
**3.00**	3.10	3.12	3.13	3.08
**5.00**	5.12	5.32	5.25	5.14
**7.00**	6.89	6.94	6.81	6.90
**9.00**	8.76	8.84	8.80	8.78
**11.00**	10.77	10.83	10.74	10.72

Note: Final pH was measured in the filtered TC adsorption solution after the selected equilibration period. If these values are means of triplicate experiments, replace each final-pH value with mean ± standard deviation. The final pH changes relative to the initial values ranged from −0.28 to +0.32 pH units.
